# Dietary Methionine Supplementation Improves Rainbow Trout (*Oncorhynchus mykiss*) Immune Responses Against Viral Haemorrhagic Septicaemia Virus (VHSV)

**DOI:** 10.3390/biology15020163

**Published:** 2026-01-16

**Authors:** Mariana Vaz, Gonçalo Espregueira Themudo, Inês Carvalho, Felipe Bolgenhagen Schöninger, Carolina Tafalla, Patricia Díaz-Rosales, Benjamín Costas, Marina Machado

**Affiliations:** 1 Centro Interdisciplinar de Investigação Marinha e Ambiental (CIIMAR), University of Porto, Terminal de Cruzeiros do Porto de Leixões, Av. General Norton de Matos s/n, 4450-208 Porto, Portugal; gthemudo@ciimar.up.pt (G.E.T.); maria.carvalho@ciimar.up.pt (I.C.); fschoninger@ciimar.up.pt (F.B.S.); bcostas@ciimar.up.pt (B.C.); mcasimiro@ciimar.up.pt (M.M.); 2 Faculty of Mathematics and Natural Sciences, University of Bergen, 5007 Bergen, Norway; 3 Instituto de Ciências Biomédicas Abel Salazar (ICBAS-UP), University of Porto, Rua de Jorge Viterbo Ferreira 228, 4050-313 Porto, Portugal; 4 Fish Immunology and Pathology Group, Biotechnology Department, National Institute for Agricultural and Food Research and Technology (INIA), Spanish National Research Council (CSIC), Carretera de La Coruña km 7.5, 28040 Madrid, Spain; tafalla@inia.csic.es (C.T.); pdiazrosales@inia.csic.es (P.D.-R.)

**Keywords:** immunomodulation, inflammation, immune response, transcriptomics, amino acids

## Abstract

Several studies have demonstrated that methionine supplementation in fish diets enhances immune status, inflammatory response, and resistance to bacterial infections by modulating for DNA methylation, aminopropylation, and transsulfuration pathways. However, the immunomodulatory effects of methionine in viral infections remain unexplored. This study aimed to evaluate the effect of methionine supplementation on immune modulation and resistance to the viral haemorrhagic septicaemia virus (VHSV) in rainbow trout (*Oncorhynchus mykiss*). Two diets were formulated and fed to juvenile rainbow trout for four weeks: a control diet (CTRL) with all nutritional requirements, including the amino acid profile required for the species, and a methionine-supplemented diet (MET), containing twice the normal requirement of DL-methionine. After feeding, fish were bath-infected with VHSV, while control fish were exposed to a virus-free bath. Samples were collected at 0 (after feeding trial), 24, 72, and 120 h post-infection for the haematological profile, humoral immune response, oxidative stress, viral load, RNAseq, and gene expression analysis. In both diets, results showed a peak in viral activity at 72 h, followed by a reduction in viral load at 120 h, indicating immune recovery. During the peak of infection, leukocytes, thrombocytes, and monocytes migrated to the infection site, while oxidative stress biomarkers (superoxide dismutase glutathione S-transferase, and glutathione redox ratio) suggested a compromised ability to manage cellular imbalance due to intense viral activity. At 120 h, immune recovery and homeostasis were observed due to an increase in the amount of nitric oxide, GSH/GSSG levels, leukocyte replacement, monocyte influx, and a reduction in the viral load. When focusing on the infection peak, gene ontology (GO) analysis showed several exclusively enriched pathways in the skin and gills of MET-fed fish, driven by the upregulation of several key genes. Genes involved in recognition/signalling, inflammatory response, and other genes with direct antiviral activity, such as TLR3, MYD88, TRAF2, NF-κB, STING, IRF3, -7, VIG1, caspases, cathepsins, and TNF, were observed. Notably, VIG1 (viperin), a key antiviral protein, was significantly upregulated in gills, confirming the modulatory role of methionine in inducing its transcription. Viperin, which harbours an S-adenosyl-L-methionine (SAM) radical domain, is directly related to methionine biosynthesis and plays a critical role in the innate immune response to VHSV infection in rainbow trout. In summary, this study suggests that dietary methionine supplementation can enhance a more robust fish immune response to viral infections, with viperin as a crucial mediator. The improved antiviral readiness observed in MET-fed fish underscores the potential of targeted nutritional adjustments to sustain fish health and welfare in aquaculture.

## Simple Summary

This study explored the impact of dietary methionine supplementation on immune modulation and resistance to the viral haemorrhagic septicaemia virus (VHSV) in rainbow trout (*Oncorhynchus mykiss*). Juvenile fish were fed for four weeks with either a control diet meeting standard amino acid requirements or a methionine-supplemented diet containing twice the normal DL-methionine level. Following infection with VHSV, samples were collected at multiple time points to assess immune responses, oxidative stress, viral load, and gene expression. Methionine supplementation influenced leukocyte and monocyte activity, oxidative balance, and the expression of antiviral genes, particularly at 72 h post-infection when viral activity peaked. Transcriptomic analysis revealed the enhanced activation of immune-related pathways in the skin and gills of supplemented fish, including the upregulation of TLR3, NF-κB, IRF3/7, and VIG1 (viperin). The strong induction of VIG1, a S-adenosylmethionine dependent antiviral protein linked to methionine metabolism, indicates a key role in host defence. Overall, methionine-enriched diets improved immune recovery and activity against the virus, suggesting that dietary methionine may act as a nutritional immunostimulant and potential future prophylactic strategy to enhance fish health and resilience in aquaculture.

## 1. Introduction

Incorporating essential amino acids into fish diets is a critical strategy to ensure proper support of the immune functions in fish. Recently, several studies have highlighted the importance of these constituents, also called functional amino acids, in terms of physiology, protein and hormonal synthesis, immune response, and oxidative stress [1]. Among functional amino acids, methionine has been shown to play a crucial role in modulating the immune system of fish, particularly when included in their diet at levels above the established requirement. Methionine is involved in processes that regulate inflammation and apoptosis, and its dietary supplementation has proven its significant potential as an immunomodulator during bacterial infections, ultimately enhancing the welfare of fish in aquaculture [2,3,4,5]. Methionine participates in three metabolic routes with direct association with the immune system: DNA methylation, aminopropylation, and transsulfuration pathways. S-adenosylmethionine (SAM) is the central compound for the above-mentioned biological processes, being synthesised in the cytoplasm of all cells through the reaction between methionine and ATP catalysed by SAM synthetase or methionine adenosyltransferase (MAT). SAM, an important biological sulphonium compound, participates in a wide range of chemical and biological processes [6]. Its primary role is to donate methyl groups to multiple metabolic pathways [5,6,7]. Methylation occurs as a modification of a proteins, DNA, and RNA to regulate gene transcription and expression. This pathway also controls the activity of various signalling pathways, such as autophagy [6]. When the transmethylation reaction occurs, SAM is converted into S-adenosylhomocysteine (SAH), a by-product that is eventually transformed into homocysteine. This molecule is a precursor involved in the synthesis of cysteine, one of the components of glutathione (GSH), through the transsulfuration pathway, especially in the liver. GSH protects against damage, helping the fish to clear damaged and toxic cells, aiding in cellular regeneration [6], and thus protecting against oxidative stress during inflammation [8]. Finally, via the aminopropylation pathway, SAM is decarboxylated, forming decarboxylated SAM (dcSAM), and uses this compound as a donor of propylamine [6], participating in the synthesis of polyamines (spermine and spermidine) [5,9]. This pathway is responsible for cellular proliferation and growth [6]. In summary, SAM plays a crucial role in cellular growth, apoptosis, death, and differentiation [6], as well as in regulating immune processes, energy metabolism, and reproduction [1,10].

Several physiological processes in fish are influenced by dietary methionine levels. Studies by Sui et al. [11] indicate that methionine availability influences growth performance, oxidative balance, and systemic metabolism in turbot (*Scophthalmus maximus*), affecting pathways such as glycolysis, lipogenesis, and fatty acid oxidation. The authors also report that dietary supplementation with crystalline methionine, up to its physiological requirement, enhances growth performance and feed efficiency. European seabass (*Dicentrarchus labrax*) fed increasing levels of methionine showed a higher response of inflammatory mechanisms, displaying an enhanced inflammatory response and resistance to *Photobacterium damselae* subsp. *piscicida* after 2–4 weeks [2,8]. Similarly, in rainbow trout (*Oncorhynchus mykiss*), doubling dietary methionine above the nutritional requirement reduced pro-inflammatory gene expression and increased peripheral neutrophil levels, confirming methionine’s immunomodulatory effects in fish [5]. Altogether, these findings suggest that methionine supplementation enhances the number of circulating neutrophils, potentially via the aminopropylation pathway, through which methionine promotes the biosynthesis of polyamines, which are essential for cell growth and proliferation, while reducing pro-inflammatory gene expression [5,7]. Additionally, as a precursor of the antioxidant molecule glutathione via the transsulfuration pathway, methionine has been described as a key factor in maintaining oxidative balance, which is involved in the regulation of cataract development in rainbow trout [12,13].

The substantial economic impact of viral outbreaks in aquaculture underscores the pressing need to develop prophylactic and therapeutic solutions. Rainbow trout production is severely affected by viral infections such as the viral haemorrhagic septicaemia virus (VHSV), which leads to high mortality rates and consequently significant economic losses [14,15,16]. There are no vaccines or completely effective treatments for this virus. VHSV belongs to the *Novirhabdovirus* genus of the Rhabdoviridae family [16,17,18] and has an enveloped negative-sense single-stranded RNA (ssRNA) virus structure, with six genes encoding nucleoprotein (N), phosphoprotein (P), matrix protein (M), glycoprotein (G), non-virion protein (NV), and RNA-dependent RNA polymerase (L). When infected with this pathogen, fish develop an innate immune response through the rapid induction and activation of type I interferon (IFN I) transcription [19], helping to control viral replication and spread by inducing an antiviral state in the fish [20]. This activation triggers the expression of interferon-stimulated genes (ISGs), namely, Mx (Myxovirus), a viperin virus-inhibitory protein associated with the endoplasmic reticulum, inducible by interferon (VIG), also known as Protein 2, containing the radical S-adenosylmethionine domain (RSAD2), interferon regulatory factors (IRFs), and interferon-stimulated gene 15 (ISG15), which are important molecules with direct action against VHSV, aiding in the reduction and degradation of viral particles [21]. Such a response was confirmed in trout by Vaz et al. [22] due to the positive and sustained action of VIG and IRFs throughout the entire course of VHSV infection in rainbow trout. Immunostimulant additives are gaining increasing appeal as promising strategies to boost fish immune systems. Thus, evaluating the ability of methionine to enhance the fish’s response to viral infections represents a crucial step forward, since no work has been conducted on the subject.

Recognising the immunomodulatory effects recently described for methionine and the beneficial outcome against bacterial infections [2,5,8], its positive action in sustaining antiviral responses is hypothesised in this study, as its role in fish remains completely unexplored. Although no studies are currently available on the matter in fish, it is known that methionine supplementation in humans improves early viral kinetics and promotes the induction of ISGs in response to the Hepatitis C virus [23]. Therefore, in the present study, the authors investigate the ability of methionine dietary supplementation to modulate in the immune response and antiviral mechanisms of rainbow trout against VHSV.

## 2. Materials and Methods

### 2.1. Experimental Diets

Two experimental diets were formulated, a control (CTRL) and a methionine diet (MET), which were produced by Sparos Lda (Olhão, Portugal). The CTRL diet was formulated to include the ideal amino acid (AA) profile according to the nutritional requirements of rainbow trout [24]. With the CTRL diet as basis, a DL-methionine supplemented diet was formulated to include double the amount of the established nutritional requirement for the species (Table 1). The diets used were the same as those used by Carvalho et al. [25].

Formulations and proximate compositions of experimental diets are presented in Table 1. All the main ingredients of the diets were ground (below 250 µm) using a micropulverizer hammer mill (SH1; Hosokawa Micron, B.V., Doetinchem, The Netherlands). The oils and powdered ingredients were then mixed according to the target formulation in a paddle mixer (RM90; Mainca, S.L., Granollers, Spain). Both diets were produced by extrusion with controlled temperature (pellet size: 1 mm) using a low-shear extruder (P55; Italplast, S.r.L., Parma, Italy). After extrusion, all batches of feed were dried in a convection oven (OP 750-UF; LTE Scientifics, Oldham, UK) for 4 h at 45 °C. Proximal composition analyses were performed using the following methodology: dry matter—by drying at 105 °C for 24 h; ash—by combustion at 550 °C for 12 h; crude protein (N × 6.25)—by flash combustion technique, followed by gas chromatographic separation and thermal conductivity detection (LECO FP428); fat—extraction with petroleum ether using the Soxhlet method; total phosphorus—according to the ISO/DIS 6491 method, using the vanado-molybdate reagent; and gross energy—determined with an adiabatic bomb calorimeter (IKA).

The two diets were also analysed for total AA profile (Table 2) by the certified laboratory Eurofins Food & Agro (Lisbon, Portugal), taking into account the ISO 13903:2005 method; EU 152/2009 (analysis performed on: 31 March 2023).

### 2.2. Feeding and Infection Trials

The study was developed at Centre of Marine and Environmental Research (CIIMAR), University of Porto, following “The guidelines on animals protection used for scientific purposes from the European Directive 2010/63/EU”, reviewed and approved by 0421/000/000/2023. The procedures were carried out by trained scientists following FELASA category C recommendations.

Juvenile rainbow trout (*Oncorhynchus mykiss*) were obtained from a commercial fish farm (Serra de Aire, Portugal). Thirty-five fish (5.9 ± 0.9 g) per tank were distributed in a total of 32 tanks (60 L) and maintained in an open system (water renewal rate, 60 L h^−1^), temperature 13.7 ± 0.7 °C, and automatically controlled photoperiod (12 h light/12 h dark) for three weeks to acclimatise. Throughout the feeding period, water parameters were recorded daily: pH 6.99 ± 0.18, dissolved oxygen 7.46 ± 0.54 mg L^−1^, and ammonia 0.26 ± 0.19 mg L^−1^.

After the acclimatization period, diets were introduced, and half of the animals were fed twice a day (2% of biomass) for four weeks with the CTRL diet, while the remaining half were fed the MET diet. Sampling was carried out after the feeding period (0 h, 1 fish per tank, and *n* = 16). Afterwards, 54 fish from each diet were collected and randomly distributed into two similar RAS systems, each consisting of 6 tanks, while the remaining fish were used for a different trial. To achieve this, 4 fish were collected from 6 of the original 16 tanks per diet, and 3 fish were collected from the remaining 10 tanks, resulting in a total of 54 fish. These 54 fish were then randomly allocated across the 6 tanks (3 for each diet), with 9 fish per tank. Afterwards, infection procedure was carried out by bath by reducing the water volume to 5 L for a period of 2 h with strong aeration and with 10^5^ TCID_50_ mL^−1^ of VHSV (strain 0771) provided by Dr. Carolina Tafalla. Sham trout were exposed to the same bath procedure but without virus. Water level was then restored to the initial 60 L. Samples were taken at 24, 72, and 120 h (3 fish per tank, *n* = 9) after bath procedure. At each sampling point, fish were euthanized (1 mL L^−1^ of 2-phenoxyethanol; Sigma-Aldrich, ref.: 77699-2.5 L, Saint Louis, MO, USA), blood was collected for haematological profile (total red and white blood cell counts, haematocrit, and haemoglobin), and plasma was obtained for humoral response. Gills and skin were also collected for RNAseq, gene expression, and viral load, while head kidney (HK), spleen, and liver were sampled for viral load. The liver was also used for biomarkers of oxidative stress.

### 2.3. Haematological Parameters and Humoral Parameters

The haematological profile of rainbow trout was analysed through blood collection from the caudal vein using heparinized syringes. The blood was stored in microtubes with 20 µL of heparin (2000 U). Red blood cell (RBC) and white blood cell (WBC) counts were determined using a Neubauer chamber. Haematocrit values were determined, and haemoglobin concentration was measured using the colorimetric method by Drabkin (SPINREACT, ref.:1001230, Girona, Spain). For the differential leucocyte count, blood smears were prepared from fresh blood, air dried, and fixed with a formaldehyde–ethanol solution, according to Kaplow and Ladd [26]. Neutrophil staining was carried out as described in Afonso et al. [27], after which, the slides were stained with haematoxylin and eosin. A total of 200 leukocytes were counted, from which the percentages of neutrophils, lymphocytes, monocytes, and thrombocytes were determined. Blood indices, including mean corpuscular volume (MCV), mean corpuscular haemoglobin (MCH), and mean corpuscular haemoglobin concentration (MCHC), were measured according to Machado et al. [2].

Then, the remaining blood was centrifuged (10,000× *g*, 10 min) to obtain plasma, which was subsequently stored at −80 °C until analysis. The activities of antiproteases and proteases, lysozyme levels, and plasma peroxidases were determined following Vaz et al. [22]. The amount of nitric oxide in the plasma was evaluated following the instructions of the NITRITE/NITRATE colorimetric method kit (Roche Applied Science, ref.: 11746081001, Basel, Switzerland). The rainbow trout plasma used was diluted 1:10 in distilled water, and a calibration curve of sodium nitrite (500 mg L^−1^) was made with serial dilutions.

### 2.4. Biomarkers of Oxidative Stress

Biomarkers of oxidative stress were measured in the liver samples of rainbow trout. The liver was homogenised with K-phosphate buffer (0.1 M, pH 7.4) in the proportion of 1 mL buffer for 100 mg tissue. After this step, the homogenised liver was centrifuged for 20 min at 10,000× *g*, 4 °C. Finally, the supernatant was collected, and the necessary aliquots were made for the oxidative biomarkers. For biomarker analysis, total protein was first quantified using the kit instructions for Pierce^TM^ BCA Protein Assay. The tissue was diluted 1:50, and then 25 µL of each sample was added in duplicate in a 96-well plate, and the optical density was read at 550 nm. The results obtained were analysed against a standard curve. The enzymatic activity of superoxide dismutase (SOD) and catalase (CAT) were determined according to Peixoto et al. [28]. Glutathione S-transferase (GST) activity in liver was determined according to the method described in Habig et al. [29] and adapted to microplate by Frasco and Guilhermino [30]. The ratio between reduced (GSH) and oxidised (GSSH) glutathione–GSH/GSSH was determined using a commercial kit (Oxford Biomedical Research, ref.: GT40, Oxford, UK) according to Reference [31]. With this method, it is possible to determine the quantities of total glutathione (GSH with GSSG) and GSSG through a microplate reading at 412 nm. The GSH/GSSG ratio is calculated as follows: (GSHt–2GSSG)/GSSG [32].

### 2.5. RNA Extraction and Quantification

RNA was extracted from the virus and fish skin, gills, liver, head kidney, and spleen following the instructions of the Maxwell^®^ RSC simplyRNA Tissue Kit (Promega, Madison, WI, USA), used with the Maxwell^®^ RSC Instruments. All RNA from tissues and virus was used for quantification of viral load, and RNA from skin and gills was also used for RNAseq and gene expression. After extraction, samples (except for sequencing) were quantified by spectrophotometry using DeNovix DS-11 FX (v4.3.8) (Wilmington, DE, USA), and its integrity was analysed with 2% agarose gel. Afterwards, RNA was converted into cDNA using the NZY First-Strand cDNA Synthesis Kit (NZYtech, Lisbon, Portugal, ref.: MB17002), for viral load and gene expression analysis.

#### 2.5.1. Virus Quantification

Viral particles’ quantification in the tissues was determined in triplicate using an iTaq^TM^ Universal Probes Supermix (Bio-rad, Hercules, CA, USA), with a final volume of 10 µL. Primers used were VHSV-N-for (5′-GACTCAACGGGACAGGAATGA–3′) and VHSV-N-rev (5′-GGGCAATGCCCAAGTTGTT–3′) and a specific probe for VHSV (TGGGTTGTTCACCCAGGCCGC) at the 5’ end with the reporter molecule 6-carboxy fluorescein (FAM) and at the 3’ end with the quencher 6-carboxytetramethyl-rhodamine (TAM) [33] at a final concentration of 10 µM. A standard curve was prepared with serial dilutions [33]. The run was performed using the CFX384^TM^ Real-Time System instrument (Bio-Rad, USA) with the following conditions: polymerase activation and DNA denaturation 10 s at 95 °C and 40 cycles of 5 s at 95 °C and 30 s at 60 °C. Threshold cycle (Ct) values were obtained, which were used to calculate the amount of virus in each sample, following the standard curve equation, [x = (yc)/m], against TCID_50_.

#### 2.5.2. Gene Ontology Functional Enrichment

The purity and concentration of each RNA sample was quantified using the Qubit^TM^ RNA Broad-Range (BR) assay kits (Thermo Fisher Scientific, ref.: Q10210, Pittsburgh, PA, USA), following the instructions. From each treatment and sampling time, random samples were selected and pooled (*n* = 3 fish per pool, *n* = 3 pools per treatment). After RNA quantification, pools were sequenced, barcoded, and sent to Novogene (Cambridge, UK) for sequencing. Upon arrival, samples were subjected to an RNA quality control protocol, and a total of 12X libraries were built using Illumina’sTruSeq stranded mRNA kit and sequenced on an Illumina Novaseq 6000 instrument as PE150 reads.

The sequencing output quality was assessed using FastQC v0.11.8 (https://www.bioinformatics.babraham.ac.uk/projects/fastqc/) (accessed on 27 March 2024) and low-quality reads (Phred quality score < 15 and read length < 30 bp) were discarded using Fastp v0.23.4 [34]. Trimmed reads were pseudo-aligned against the rainbow trout reference transcriptome (USDA_OmykA_1.1) using Kallisto v0.46.0 [35]. Transcript level expression was imported to R v4.2.1 using R/tximport v1.26.1. Comparisons were made against the CTRL 0 h data—non-infected—which were used as the reference. Genes with False Discovery Rate adjusted *p*-values ≤ 0.01 and an absolute Log2 Fold Change (Log2FC) ≥ 2 in relation to the CTRL 0 h group were considered differentially expressed genes (DEGs).

These DEGs served as inputs for gene ontology (GO) analysis in g:Profiler (version e111_eg58_p18_f463989d, https://biit.cs.ut.ee/gprofiler/gost (accessed on 27 March 2024)). GO terms were analysed and categorised as biological processes (BP), cellular components (CC), and molecular functions (MF) using the Benjamini–Hochberg FDR (*p*-values < 0.05 were considered enriched). Of the total enriched GO terms, all those related to immunities and/or responses to viruses were selected. The Venn diagrams (Figures S1 and S2 of the Supplementary Material—Venn) were made using the online server https://asntech.shinyapps.io/intervene/ (accessed on 27 March 2024). The bubble charts presented are results of the enriched GO terms selected and were constructed using the RStudio software (version R 4.2.1). The gene names within the enriched GO terms were generated using the Ensembl BioMart tool (https://www.ensembl.org/index.html (accessed on 27 March 2024)). Relevant information, including the best-matching protein sequences, query cover, total score, and maximum score, were retrieved from BLAST (2.16.0) results (available at repository DOI: 10.6084/m9.figshare.30209632).

#### 2.5.3. Gene Expression

Quantitative PCR was performed on the CFX384^TM^ Real-Time System (Bio-Rad, USA), using 0.86 µL of skin cDNA and 0.53 µL of gills cDNA (already diluted, considering a maximum of 20 ng per well), mixed with 5 µL of NZYSupreme qPCR Green Master Mix (2x) (NZYtech, Lisbon, Portugal, ref.: MB41903) and 0.3 µL of each primer (10 µM), with a final volume of 10 µL. Standard cycle conditions were 95 °C for initial denaturation for 3 min, followed by 40 cycles of 95 °C for 30 s for denaturation and annealing/extension for 20 s, with temperature-specific conditions for each set of primers (Table 3), and 72 °C final extension for 30 s. All samples were analysed in duplicate.

Immune genes were selected (Table 3) based on results obtained in RNAseq, including Toll-like receptor 3 (*tlr3*), Myeloid differentiation primary response 88 (*myd88*), stimulator of interferon genes protein-like (*sting*), interferon regulatory factor 3 and 7 (*irf3* and *irf7*), viperin (*vig1*), and tumour necrosis factor (*tnfα*). Some primers were designed using NCBI Primer Blast Tool according to known qPCR restrictions (amplicon size, Tm difference between primers, GC content, and self-dimer or cross-dimer formation). All reactions were carried out in duplicate. The target gene means were normalised using the delta-delta Ct method. The housekeeping genes, β-actin (*actβ*), Elongation factor 1-alpha (*ef1α*), and 18 S ribosomal RNA (*18s*), were used for gene normalisation.

### 2.6. Data Analysis

Except for RNAseq, all results are presented as mean ± standard deviation (mean ± SD). The obtained data were analysed considering normality and homogeneity of variances through the Shapiro–Wilk and Leven’s test, respectively, and transformed, if necessary, before statistical treatment.

One-way ANOVA was performed to investigate the effects of the diets on the fish’s immune statuses after 4 weeks of the feeding trial (T0h), and a three-way ANOVA was performed to explore the effects of diet and time on the early response to VHSV. Whenever diet or time was significant, Holm–Šídák post hoc test was performed to identify differences among groups within the variables (time post-infection × infection × diet). Statistical significance was tested at *p*-value < 0.05, and all statistical analyses were performed using SigmaPlot 12.0 software for Windows.

## 3. Results

### 3.1. Haematological Parameters and Differential Leukocyte Counts

Table 4 presents the values of the haematological parameters analysed in rainbow trout fed CTRL and MET diets for 4 weeks and posteriorly bath-challenged with VHSV or a sham solution. No statistically significant differences were observed between the CTRL and MET groups immediately after the feeding period (0 h). Irrespective of the bath solution (with or without virus), the modulation of MCV and MCHC values was observed, with a decrease over time in the MCV values and a drop followed by a recovery of the MCHC ratio at 72 h post-infection. Diet also modulated fish WBC concentration at 120 h post-bath procedure and irrespective of infection treatment, with MET dietary treatment showing reduced levels at 120 h compared to fish fed the CTRL diet. Additionally, WBC numbers were also found to be significantly reduced in infected animals at 72 h while significantly increased at 120 h compared to non-infected animals. Non-infected animals also display higher values of RBC and HT at 120 h for both parameters and 72 h for HT only. Finally, MCH values are found to be significantly decreased at 72 h onwards in all treatments. Table S1, included in the Supplementary Materials, details the statistical differences obtained.

**Figure 1:**
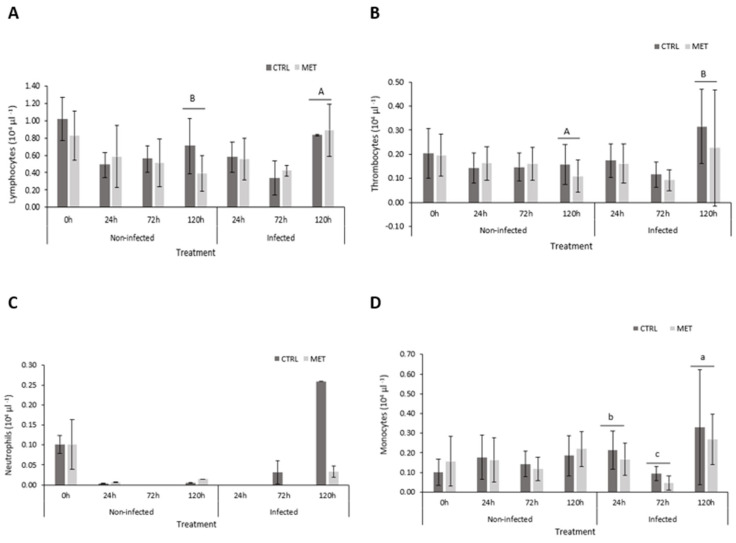
Differential leukocytes in the blood of rainbow trout fed the CTRL and MET diets, exposed to a challenge with VHSV, and sampled at 0, 24, 72, and 120 h post-infection. (**A**) Lymphocytes; (**B**) thrombocytes; (**C**) neutrophils; and (**D**) monocytes. Values represent means ± SD (*n* = 16 for 0 h; *n* = 9 for 24, 72, and 120 h). Different lowercase letters indicate differences between times (0, 24, 72, and 120 h), and uppercase letters indicate differences between infection (non-infected vs. infected). Different symbols represent differences between experimental diets (CTRL vs. MET). Multifactorial ANOVA; Holm–Šídák post hoc test; *p* ≤ 0.05. CTRL (control diet), MET (methionine diet).

Looking at fish differential leukocyte counts (Table S2, Supplementary Materials), no significant differences were observed between the diets neither after the 4-week feeding period nor in response to viral challenge. Nonetheless, the number of circulating lymphocytes and thrombocytes (Figure 1A, B, respectively) of rainbow trout varied according to time and infection, with a significantly higher concentration of both cells in infected fish at 120 h compared to uninfected individuals. Moreover, a peak in both lymphocytes and thrombocytes was observed in infected fish at 120 h. In terms of neutrophils (Figure 1C), no significant differences were found between groups, but note that at 0 h, the number of circulating neutrophils is higher when observing the non-infected remaining groups. Finally, in response to the virus, monocytes (Figure 1D) were found to be decreased at 72 h, followed by a peak in their number at 120 h.

### 3.2. Humoral Immune Parameters

**Figure 2:**
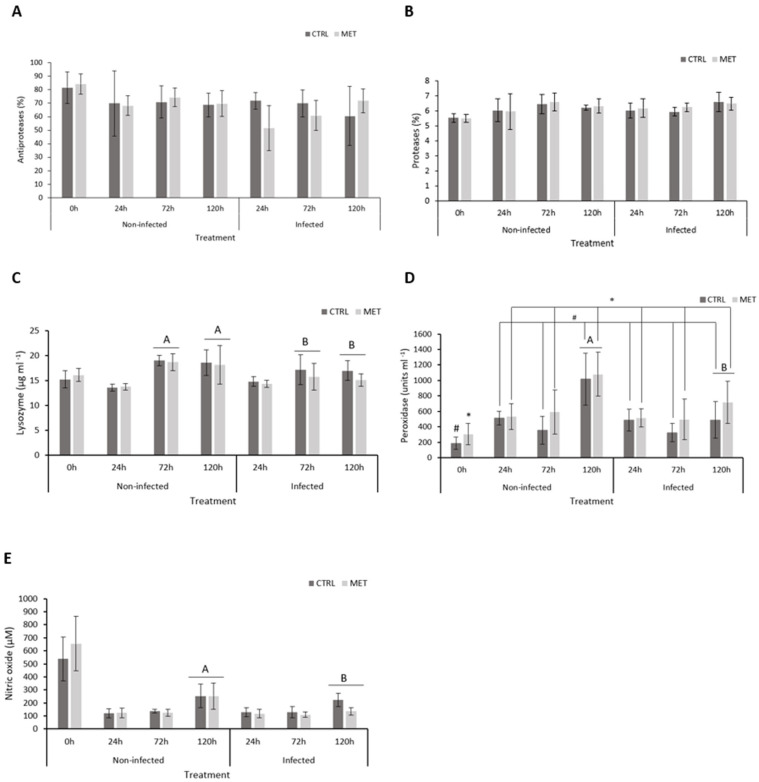
Immune response parameters analysed in the plasma of rainbow trout fed with the CTRL and MET diets, exposed to a VHSV challenge, and sampled at 0, 24, 72, and 120 h after infection: (**A**) antiproteases activity, (**B**) proteases activity, (**C**) lysozymes, (**D**) peroxidase activity, and (**E**) nitric oxide concentration. Values represent means ± SD (*n* = 16 for 0 h; *n* = 9 for 24, 72, and 120 h). Different uppercase letters indicate differences between infection (non-infected vs. infected). Different symbols (* or #) represent differences between experimental diets (CTRL vs. MET). Multifactorial ANOVA; Holm–Šídák post hoc test; *p* ≤ 0.05. CTRL (control diet), MET (methionine diet).

The innate immune response was evaluated in rainbow trout plasma after the feeding period and during the different times of infection (Figure 2, Table S3, Supplementary Materials). The antiproteases and proteases activity (Figure 2A,B, respectively) showed no significant changes throughout the trial. No effect of the diet was visible for lysozyme (Figure 2C) and nitric oxide concentrations (Figure 2E); nonetheless, they varied with time after infection and infection state. A significant increase in the plasma lysozyme from 24 h to 72 h was observed in both uninfected and infected animals, with non-infected individuals presenting higher values at 72 h and 120 h than infected ones. Peroxidase activity (Figure 2D) varied between diets immediately after four weeks of feeding (0 h), with the MET diet significantly higher than the CTRL diet. This pattern appears between the MET and CTRL diets, regardless of the type of infection, throughout the entire trial. An interaction between time and infection was also observed for this enzyme, with a reduction in the infected group at the last sampling time. The latter was also observed for plasma nitric oxide at 120 h (Figure 2E), being that this time point was generally higher than the previous sampling points.

### 3.3. Biomarkers of Oxidative Stress

**Figure 3:**
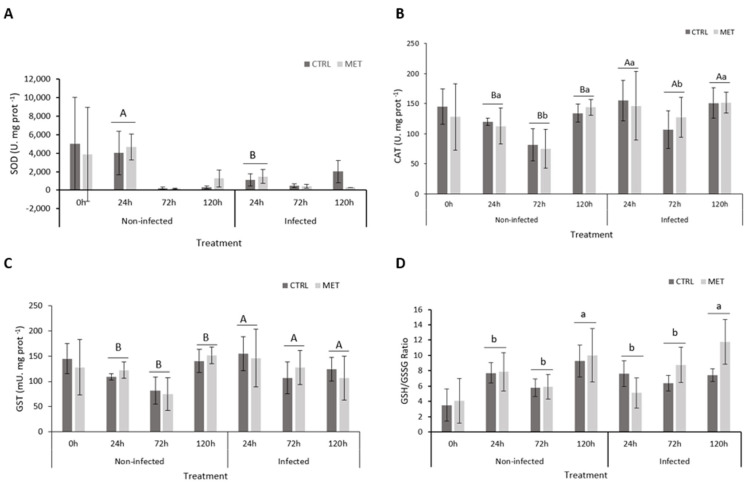
Oxidative stress biomarkers analysed in the liver of rainbow trout fed the CTRL and MET diets, exposed to a challenge with VHSV, and sampled at 0, 24, 72, and 120 h after infection. (**A**) Superoxide dismutase (SOD), (**B**) catalase (CAT), (**C**) glutathione S-transferase (GST), and (**D**) reduced (GSH)/oxidised glutathione (GSSG) ratio. Values represent means ± SD (*n* = 16 for 0 h; *n* = 9 for 24, 72, and 120 h). Different lowercase letters indicate differences between times (0, 24, 72, and 120 h), and uppercase letters indicate differences between infection (non-infected vs. infected). Different symbols represent differences between experimental diets (CTRL vs. MET). Multifactorial ANOVA; Holm–Šídák post hoc test; *p* ≤ 0.05. CTRL (control diet), MET (methionine diet).

No dietary effect was verified for the oxidative stress parameters analysed (Table S4, Supplementary Materials). Although not statistically significant, both un- and infected groups present a similar temporal trend for all oxidative markers, characterised by a higher response at 24 h followed by a decrease at 72 h and a subsequent increase at 120 h. This is specifically observed for SOD (Figure 3A) in the non-infected group, for catalase (Figure 3B) in both infection treatments, and for both GST and the GSH/GSSG ratio (Figure 3D) in non-infected groups. The scenario slightly changes in infected animals, with a decrease in GST (Figure 3C) and an increase in the GSH/GSSG ratio with time. Despite this time effect, SOD activity was significantly lower in infected fish at 24 h, while catalase was continuously higher in infected fish at all sampling points compared to their non-infected counterparts. At 24, 72, and 120 h, infected groups presented higher GST concentration.

### 3.4. Virus Quantification

**Figure 4:**
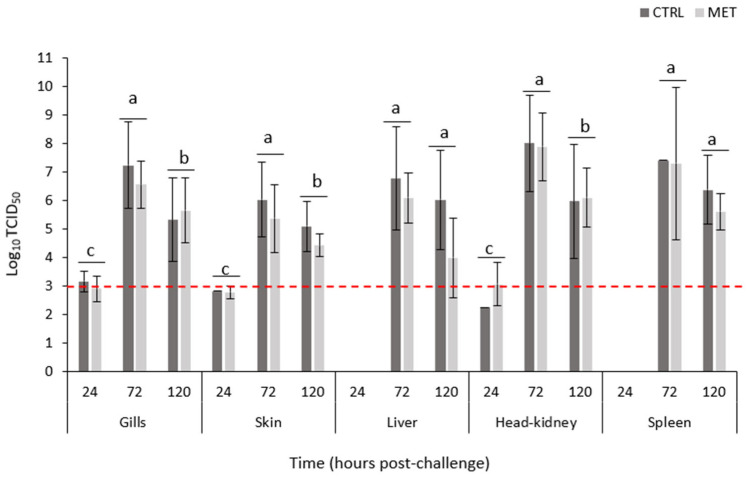
Viral load analysed in the gills, skin, liver, head kidney, and spleen of rainbow trout fed the CTRL and MET diets, exposed to a challenge with VHSV, and sampled at 0, 24, 72, and 120 h post-challenge. Different lowercase letters indicate differences between times (0, 24, 72, and 120 h). Red line is a baseline limiting the values considered virus-free (Ct values between 35 and 38), following the study by Vaz et al. [22]. Multifactorial ANOVA; Holm–Šídák post hoc test; *p* ≤ 0.05. CTRL (control diet), MET (methionine diet).

Viral load (Figure 4, Table S5, Supplementary Materials) was analysed in the gills, skin, liver, head kidney, and spleen of rainbow trout fed the CTRL and MET diets at 24, 72, and 120 h post-challenge with VHSV. In order to do so, a standard curve, prepared using serial dilutions of VHSV cDNA, was used. The determination of the viral particle quantity in the tissues was obtained using the equation Y = −3.1357x + 45.975, with R^2^ = 0.9909, following Vaz et al. [22].

No viral load was detected in non-infected individuals at any of the time points nor across the diets. Moreover, no effect of the diet was observed. At the early sampling point (24 h), viral particles were still absent in the liver and spleen, while present, despite being below the baseline and therefore considered virus-free, in the gills, skin, and head kidney. In all tissues, the peak of infection was recorded at 72 h, with a significantly higher viral load compared to the previous sampling point. In the gills, skin, and head kidney, a significant reduction in the VHSV load was observed at the final time point—120 h. On the other hand, liver and spleen viral loads remained significantly elevated after 72 h. Although not statistically significant, 72 h post-infection, the MET diet tended to have lower viral particle quantities in all tissues compared to the CTRL diet. No mortality was recorded during the trial.

### 3.5. RNAseq

#### 3.5.1. Differential Expression Genes

**Figure 5:**
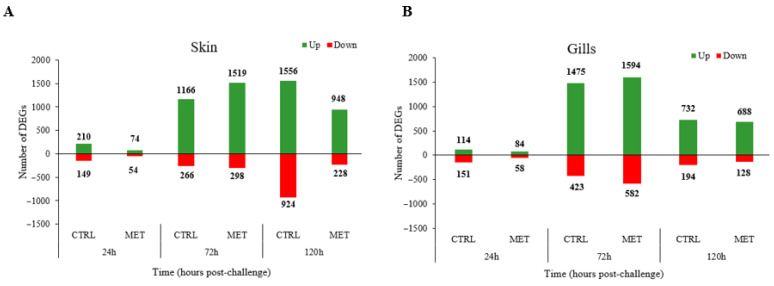
Differentially expressed genes (DEGs) upregulated and downregulated in the skin (**A**) and gills (**B**) of rainbow trout infected with VHSV, fed the CTRL and MET diets, and sampled at 24, 72, and 120 h post-challenge. CTRL (control diet), MET (methionine diet).

Based on the results, the number of both upregulated and downregulated differentially expressed genes (DEGs) in infected rainbow trout was higher in the gills (Figure 5B) than in the skin (Figure 5A) at 72 h. At 24 h, in general, there was a reduced number of upregulated and downregulated genes compared to the other time points, with a stronger response of both up- and downregulated DEGs at 72 h. Finally, at 120 h post-challenge, the number of DEGs (both upregulated and downregulated) in the skin increased under the CTRL diet compared to the number at 72 h. In contrast, at that time, the number of DEGs decreased in the gills for both diets, as well as in the skin under the MET diet.

#### 3.5.2. Gene Ontology Enrichment Analyses

**Figure 6:**
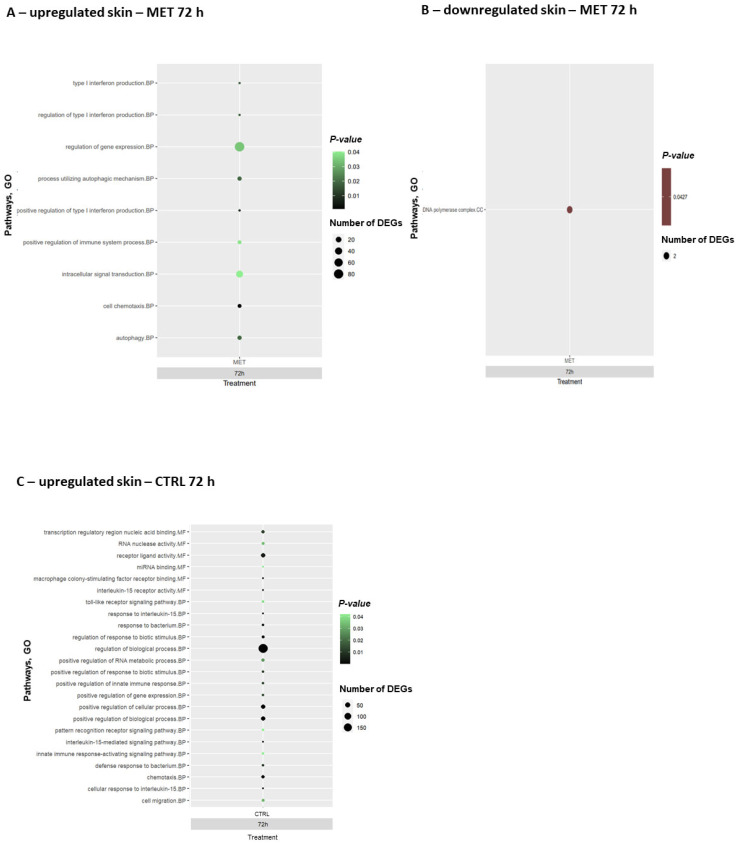
Bubble charts of gene ontology (GO) enrichment analysis in skin: (**A**) upregulated MET diet, (**B**) downregulated MET diet, and (**C**) upregulated CTRL diet in rainbow trout at 72 h post-challenge with VHSV. The pathways were categorised in terms of functions. BP: biological process; CC: cellular component; and MF: molecular function.

**Figure 7:**
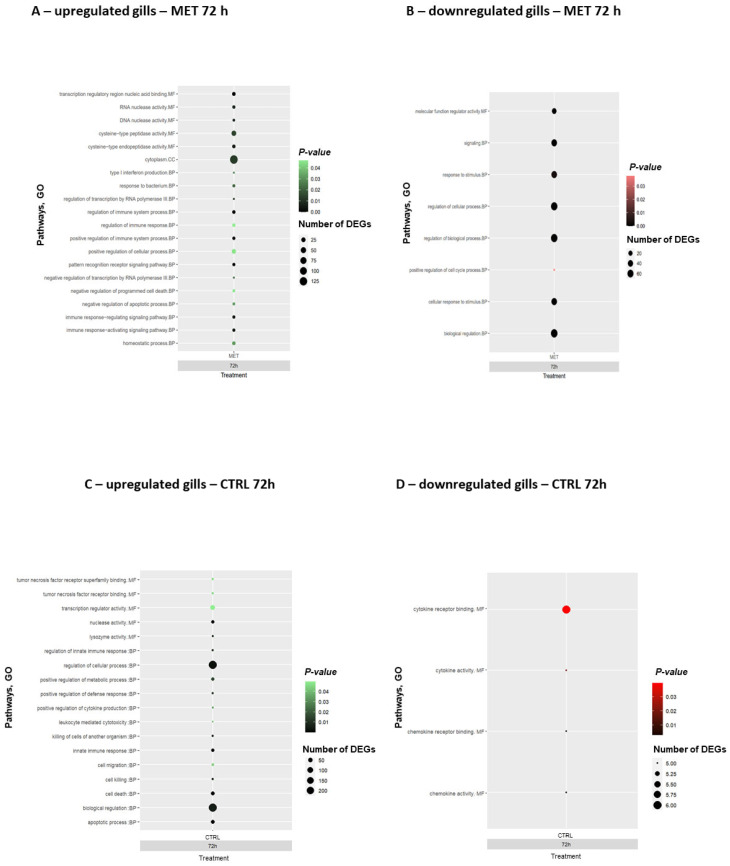
Bubble charts of gene ontology (GO) enrichment analysis in gills: (**A**) upregulated MET diet, (**B**) downregulated MET diet, (**C**) upregulated CTRL diet, and (**D**) downregulated CTRL diet in rainbow trout at 72 h post-challenge with VHSV. The pathways were categorised in terms of functions. BP: biological process; CC: cellular component; and MF: molecular function.

To understand the biological functions, cellular processes, and molecular components associated with the DEGs detected at the peak of infection (72 h), gene ontology (GO) enrichment analysis was performed when comparing the infected fish with the control diet at 0 h (after the feeding trial). Both upregulated and downregulated DEGs in the CTRL and MET diets in the skin and gills (Figure 6 and Figure 7, respectively; Supplementary Material Figures S1 and S2, respectively) were analysed.

At the peak of infection, trout skin from fish fed the MET diet showed positively regulated terms (Figure 6A), all categorised as biological processes (BP), highlighting the “positive regulation of immune system process”, “intracellular signal transduction”, “regulation of gene expression”, and three different pathways related to type I interferon production, such as the “regulation/positive regulation of type I production”. On the other hand, in this tissue, only one cellular component (CC) pathway was observed to be downregulated (Figure 6B), characterised as the “DNA polymerase complex”. The CTRL diet, on the other hand, only presented positively regulated pathways (Figure 6C), specifically the “macrophage colony-stimulating factor receptor binding” and “interleukin-15 receptor binding”, which are involved in molecular functions (MFs). In terms of biological processes, GO terms such as “toll-like receptor signalling pathway”, “response to interleukin/bacteria”, “positive regulation of gene expression”, and various pathways associated with the regulation of biological and cellular processes, as well as immune responses, were observed. In the skin, overall, the CTRL diet presented a higher number of positively regulated pathways compared to the MET diet (Supplementary File, Figure S1). In skin with the downregulated CTRL diet, no enriched GO terms were observed.

The enriched pathways driven by the upregulated DEGs in the gills of fish fed the MET diet (Figure 7A) indicated a generally stronger immune response compared to the skin. The MET diet promoted the significant positive regulation of several pathways involved in molecular functions (MFs), such as “transcription regulatory region nucleic acid binding” and “cysteine-type peptidase/endopeptidase activity”. According to the bubble chart, it is clear that the “cytoplasm” pathway, categorised as a cellular component (CC), is the most significantly enriched with the highest number of DEGs. In terms of biological processes, the greatest number of different pathways was observed, with particular attention paid to the significantly enriched ones, such as “type I interferon response”, “regulation/positive regulation of immune system process”, “positive regulation of cellular process”, “pattern recognition receptor signalling”, and “immune response-activating or regulating signalling”. The downregulated pathways in gills in response to MET supplementation (Figure 7B) are linked to the regulation of the cell cycle, response to stimulus, and biological processes. Regarding positive regulation in the CTRL diet (Figure 7C), pathways involved in “nuclease activity” (MF), “regulation of cellular process”, “positive regulation of metabolic process”, “biological process”, “killing of cells of another organism”, and several related to cell death were significantly expressed. Finally, the gills of fish fed the CTRL diet showed the downregulation of pathways related to chemokines and cytokines (Figure 7D; Supplementary File, Figure S2). The downregulated genes in the MET diet are part of a general inflammatory response (Figure 6B and Figure 7B).

**Figure 8:**
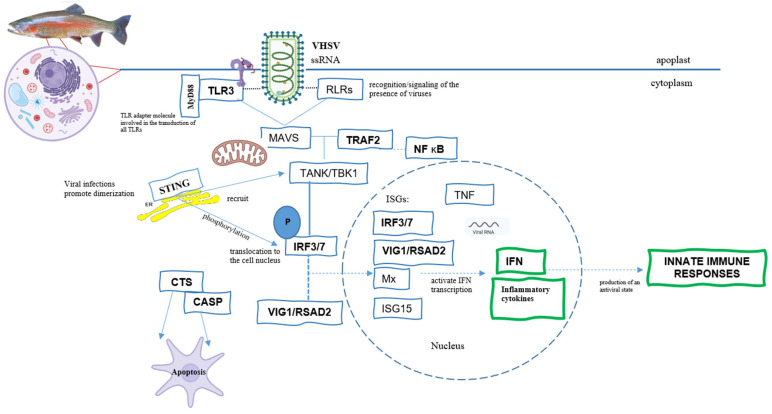
Schematic representation of the innate immune response of rainbow trout against a VHSV infection, considering the literature and the genes that were significantly more expressed in the MET diet at the peak of infection (72 h) in both tissues. The genes underlined with the darker shade (TLR3, MyD88, TRAF2, NF κB, STING, IRF3 and 7, VIP/RSAD2, CTS, CASP, and TNF) represent those that were significantly more expressed. Legend: ssRNA: single-stranded RNA viruses, TLRs: Toll-like receptors, MyD88: Myeloid differentiation primary response 88, RLRs: RIG-I-like receptors, MAVS: mitochondrial antiviral-signalling protein, TRAF2: tumour necrosis factor (TNF) receptor associated 2, TANK/TBK1: TRAF family member associated NF-kappa-B activator/TANK-binding kinase 1, NF κB: nuclear factor kappa B, STING: stimulator of interferon genes, IRF: interferon regulatory factor, ISGs: interferon-stimulated genes, VIG1/RSAD2: viperin/Radical S-Adenosyl Methionine Domain Containing 2, Mx: Myxovirus resistance, CTS: cathepsin, CASP: caspase, TNF: tumour necrosis factor, and IFN: interferon.

Elucidating methionine’s potential immunomodulatory functions in the host antiviral response in both tissues at the peak of infection (72 h), a selection of genes and GO terms exclusively upregulated in response to MET can be found in Table 5 and Figure 8. *Tlr3* and *myd88* were expressed in both tissues, skin and gills, contributing to the enrichment of the pathways: regulation of immune response and immune response-activating/regulating signalling pathway, respectively. *Traf2* and *nfkb* were only detected in the skin—“intracellular signal transduction” and “regulation of gene expression”, respectively. The sting genes, *irf3*, and *irf7*, were observed in both tissues but in distinct pathways. In the case of *sting*, pathways included type I interferon, the regulation of the immune/cellular system in gills, and type I interferon production/regulation, the regulation of gene expression in skin. Both *irf3* and *irf7* genes were regulated in the same pathways: transcription regulatory region nucleic acid binding, positive regulation of cellular process (gills), and the regulation of gene expression (skin). The positive expression of the genes *vig1/rsad2* and *tnf* was only observed in the gills in the pathways of “cytoplasm and regulation of immune response” and “positive regulation of cellular process”, respectively. The genes caspase and cathepsin were only significantly expressed in the gills in both the “cysteine-type peptidase/endopeptidase activity” pathways, with cathepsin also observed in the “cytoplasm”.

### 3.6. Gene Expression

**Figure 9:**
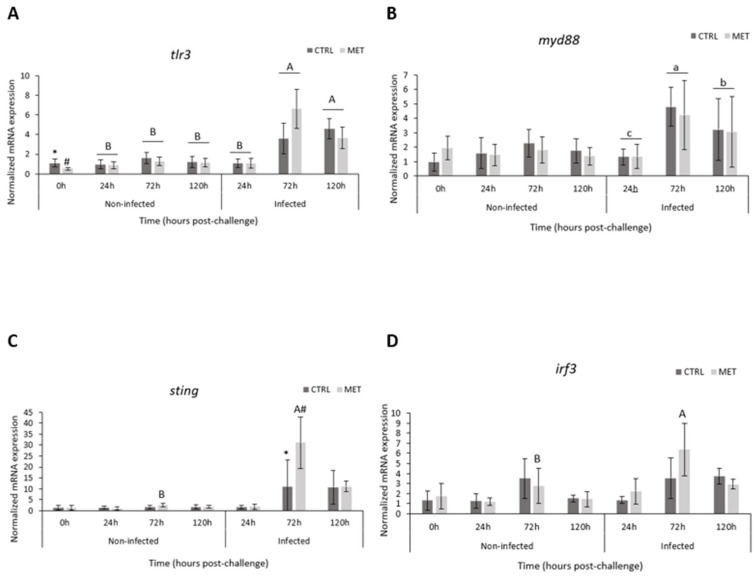

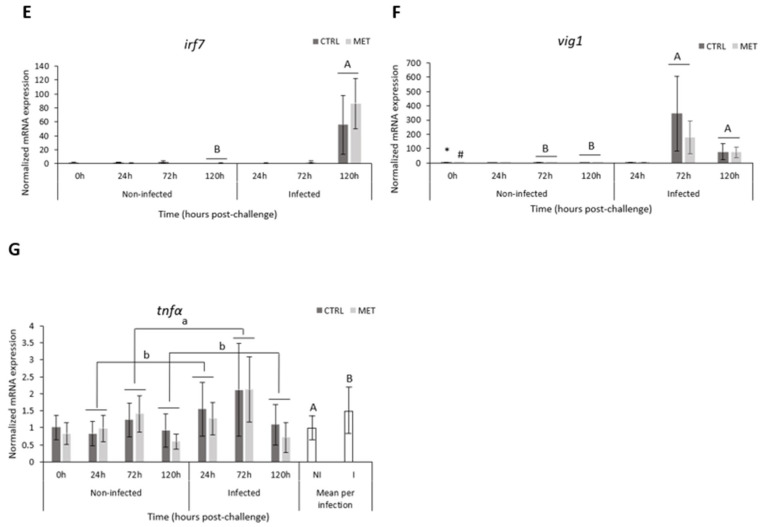
Gene expression in the skin of rainbow trout fed the CTRL and MET diets, exposed to a challenge with VHSV, and sampled at 0, 24, 72, and 120 h post-infection. (**A**) Toll-like receptor 3 (*tlr3*); (**B**) Myeloid differentiation primary response 88 (*myd88*); (**C**) stimulator of interferon genes (*sting*); (**D**) interferon regulatory factor 3 (*irf3*); (**E**) interferon regulatory factor 7 (*irf7*); (**F**) viperin (*vig1*); and (**G**) tumour necrosis factor α (*tnfα*). Values represent means ± SD (*n* = 16 for 0 h; *n* = 9 for 24, 72, and120 h). Different lowercase letters indicate differences between times (0, 24, 72, and 120 h), and uppercase letters indicate differences between infection (non-infected vs. infected). Different symbols (* or #) represent differences between experimental diets (CTRL vs. MET). Multifactorial ANOVA; Holm–Šídák post hoc test; *p* ≤ 0.05. CTRL (control diet), MET (methionine diet).

**Figure 10:**
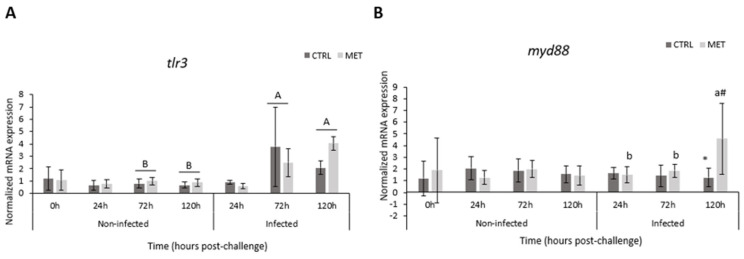

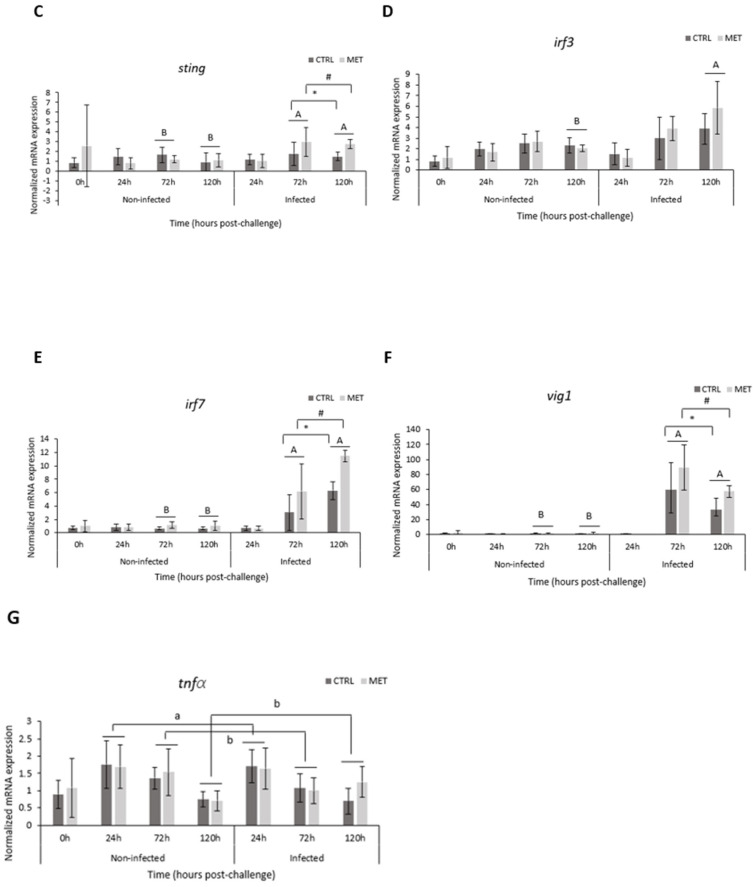
Gene expression in the gills of rainbow trout fed the CTRL and MET diets, exposed to a challenge with VHSV, and sampled at 0, 24, 72, and 120 h post-infection. (**A**) Toll-like receptor 3 (*tlr3*); (**B**) Myeloid differentiation primary response 88 (*myd88*); (**C**) stimulator of interferon genes (*sting*); (**D**) interferon regulatory factor 3 (*irf3*); (**E**) interferon regulatory factor 7 (*irf7*); (**F**) viperin (*vig1*); and (**G**) tumour necrosis factor α (*tnfα*). Values represent means ± SD (*n* = 16 for 0 h; *n* = 9 for 24, 72, and 120 h). Different lowercase letters indicate differences between times (0, 24, 72, and 120 h), and uppercase letters indicate differences between infection (non-infected vs. infected). Different symbols (* or #) represent differences between experimental diets (CTRL vs. MET). Multifactorial ANOVA; Holm–Šídák post hoc test; *p* ≤ 0.05. CTRL (control diet), MET (methionine diet).

Several genes that were significantly upregulated in the MET diet group in both skin and gill tissues (Figure 9 and Figure 10, respectively) at 72 h post-infection, as identified by RNA sequencing, were selected for further analysis. Their relative expression levels were examined over time (0, 24, 72, and 120 h) in animals fed either the CTRL or MET diet, under both sham and infected conditions (Tables S6 and S7, included in the Supplementary Materials, show the statistical differences in skin and gills, respectively). Notably, all genes analysed in the skin and gills exhibited positive expression levels.

#### 3.6.1. Skin

The regulation of *tlr3* (Figure 9A) showed changes between diets at 0 h, with the CTRL diet being significantly higher than the MET diet. Both at 72 h and 120 h during infection, they presented significantly higher values than uninfected animals, with the peak expression of this gene observed at 72 h. *Myd88* (Figure 9B) increased significantly at the peak of infection—72 h—then decreased but without reaching sham levels, which was not significant. During the final hours of infection, the expression of this gene was higher compared to virus-free fish. The regulation of *sting* (Figure 9C) remained low in uninfected animals and also 24 h after infection. Furthermore, 72 h after infection, significantly higher values were observed, with a notable increase in the MET diet. In the presence of the viral pathogen, *irf3* (Figure 9D) increased at 72 h but only in the MET diet group compared to the uninfected animals. A downward trend was also observed at 120 h. *Irf7* (Figure 9E) showed low normalised expression values up to 72 h post-infection, with a very significant increase at 120 h in the VHSV-exposed group. Of all the genes analysed, the expression of *vig1* (Figure 9F) showed significantly higher values, focusing on 72 h post-infection. After this time, a reduction in expression was observed. In uninfected fish, after the feeding period (0 h), vig1 was significantly higher in the CTRL diet. The expression of *tnfa* (Figure 9G) showed a general increase during infection regardless of diet, peaking at 72 h. Overall, *tnfa* expression in the skin of the group exposed to VHSV was significantly higher than that of the uninfected group.

#### 3.6.2. Gills

In the gills, no genes showed differences between the diets soon after the 4-week feeding period. After 72 h of exposure to VHSV, the expression of *tlr3* (Figure 10A) increased significantly compared to the sham group. In infected rainbow trout, VHSV induced a significant increase in *myd88* in the MET diet at 120 h post-infection compared to the CTRL diet and also compared to the other time points (Figure 10B). Significantly higher *sting* upregulation (Figure 10C) was observed in the presence of the virus, coinciding with the peak of infection at 72 h and higher in the MET diet group. *Irf3* (Figure 10D) tended to increase over time in infected animals, with a significantly higher expression in the presence of VHSV at 120 h. At 72 and 120 h, *irf7* (Figure 10E), in the presence of infection, showed a significant increase in MET expression compared to the CTRL diet group. Note that this expression, at the same time points, was higher than that of the sham group. *Vig1* expression (Figure 10F) at 72 h in virus-exposed fish showed a very significant increase in levels, with the MET diet showing significantly higher expression. A decrease in expression was observed at 120 h, but the MET diet group still showed a higher expression compared to the CTRL. At the last two sampling points, *vig1* was higher in the infected group. The upregulation of *tnfα* (Figure 10G) only varied over time. The presence of VHSV promoted an increase in its expression within 24 h, followed by a constant decrease until the final moment.

## 4. Discussion

In recent years, the immunomodulatory role of methionine in fish diets has been the subject of numerous studies, with its supplementation providing clear benefits to the fish’s immune systems. Specifically, it improves immune cell status and disease resistance to bacteria [2,4,8,38] and enhances neutrophil proliferation, while reducing pro-inflammatory gene expression in the absence of immune stimuli [5], thus contributing to the welfare of aquaculture. However, the studies available in the literature on the modulatory capacity of methionine only refer to either feeding trials or feeding versus bacterial infections. Given what is known about this amino acid, the present study allowed for the investigation of methionine’s ability to modulate rainbow trout antiviral responses to VHSV when fed a diet supplemented with twice the nutritional requirement.

### 4.1. Peak of Infection

To comprehensively assess the pathogen’s ability to invade host tissues, viral quantification was conducted across multiple external and internal organs. Notably, viral cDNA copies were detected as early as 24 h post-infection in the gills, skin, and HK. However, previous research indicates that viral loads around 10^3^ copies µL^−1^—as verified in the present study and corresponding to Ct values between 35 and 38—are more likely to be indicative of free virus particles rather than active replication [39]. Importantly, the infection appears to peak at 72 h post-infection, as evidenced by significantly higher viral loads compared to those observed at 24 h, followed by a marked decline by 120 h in the gills, skin, and HK. Interestingly, elevated viral loads continue in the liver and spleen at 120 h, suggesting potential differences in the viral persistence or tissue-specific dynamics. This trend aligns with findings from Vaz et al. [22].

### 4.2. Systemic Response to VHSV Infection

The haematological profile of fish immediately after the feeding period with the experimental diets or even during the viral challenge showed few changes. In response to viral infection, a drop in the peripheral WBC count and haematocrit percentage are perceived early at 72 h, followed by an increase at 120 h. The displayed leukopenia at 72 h could be a direct indication of the high viral activity observed at the peak of infection, causing these cells to migrate to the affected tissues [40,41]. In contrast, at the peak of infection (72 h) and regardless of diet, the number of RBC increased, suggesting the involvement of these cells in the antiviral response to VHSV, as nucleated red blood cells of the fish have been reported to participate in innate immune mechanisms [42,43]. Over the course of the infection, particularly at 120 h, a recovery in measured values was observed, namely, a reduction in erythrocytes and haematocrit level to baseline levels comparable to those at 0 h. This recovery overlaps with the marked reduction in viral particles in the tissues analysed.

During inflammation caused by a pathogenic agent, various physiological alterations occur, leading to the production and recruitment of surrounding leukocytes to the affected sites. Among these cells, neutrophils are the first to be recruited in response to stimuli [8], followed by monocytes, lymphocytes, and thrombocytes [22,40,41], which migrated during the most critical time of infection. In the present study, rapid neutrophilia and monocytosis were not observed when compared to undisturbed fish (0 h). In fact, a clear response of these phagocytic cells was only perceived at 120 h post-infection, with significant monocytopenia at 72 h, followed by monocytosis at 120 h, while neutrophils failed to show significant differences. This clear increase in the cellular response of monocytes at a later sampling point also occurred in the study by Vaz et al. [22]. These results were accompanied by an increase in lymphocytes and thrombocytes. Such cellular responses could be interpreted by their role in neutralising foreign bodies [40] and attempts to promote homeostasis. This coincides with the increase in lymphocytes at 120 h after exposure to VHSV, which was statistically higher than in the non-infected group, indicating that in the early hours of infection, there was a cellular imbalance [41,44].

As allies of the cellular component, antioxidant and antimicrobial mechanisms function as key defensive components against the infection agent and inherent oxidative stress during inflammatory responses [41,45,46,47]. A similar modulation of key humoral mechanisms was observed in both uninfected and infected trout, with both lysozyme and nitric oxide concentrations increasing over time after the bath challenge, clearly displaying that the handling itself could trigger a response of such enzymes, with few significant differences induced by the antigen–VHSV presence. Additionally, results seem to corroborate the observed delayed cellular response hypothesis due to an early cellular influx from the blood stream to the primary affected organs or even the immune depression at the beginning of the infection (24 h) [41,44]. This same scenario of unbalanced or delayed systemic immune response was observed by the authors during a VHSV infection in rainbow trout, ultimately supporting the hypothesis that the host has an inability to promote, in the first hours of infection, an efficient cellular production and to rapidly recognise and act against the virus [22]. At the last time point, nitric oxide levels increased as an attempt to inhibit the virus [41,48] and to promote a certain cellular balance, also justified by the reduction in viral load at this time, as its production is mainly carried out by monocytes [2,49]. The action of this compound works as a pro-inflammatory response to cytokines, namely, the tumour necrosis factor involved in bacterial lipopolysaccharide, parasites [50], and viral infections [51]. Similar observations were described by Vaz et al. [22] using the same virus and fish species, by Vaz et al. [41] using nervous necrosis virus in *Dicentrarchus labrax*, and by Ferreira et al. [52] with the same fish and a *Tenacibaculum maritimum* infection. Curiously, infected fish showed lower activity of both mechanisms at 72 h, described as the infection’s peak, and at 120 h.

Moreover, exposure to a pathogen causes stress, which tends to lead to the increased production of reactive oxygen species (ROS), leading to tissue lipid peroxidation [53]. This can damage important molecules and the fish’s own antioxidant defence system [54]. As a response to cellular stress, SOD, CAT, and GSH are important antioxidant defences responsible for removing ROS [54,55], and if their action is insufficient, lipid peroxidation may occur [56]. Early in the infection, the SOD activity significantly decreased in response to VHSV, reflecting a reduction in the ability to cope with oxidative stress [41,47]. On the other hand, CAT activity similarly varied in the uninfected and infected groups, and together with the GST activity, both decreased after 72 h of infection. These results suggest that, despite the lack of a strong response from oxidative enzymes during the challenge, the ratio of reduced oxidised glutathione increased at 120 h in both diets, indicating that, at this time, the host managed to maintain some hepatic oxidative balance, which could translate into reduced oxidative stress and tissue damage [57].

During the infection, whether in the skin or gills, there was a noticeably higher expression of most of the genes analysed by qPCR compared to the virus-free group. Interestingly, and in line with some of the results presented earlier, low or undetectable expression of the analysed genes at 24 h are followed by an increase in expression at the peak of infection (72 h) and at 120 h. In the skin, all analysed genes responded to the infection, with a marked increase in expression at the peak of VHSV activity. In contrast, in the gills, only *tnfα* showed a regulation pattern similar to that of the non-infected group. In both tissues, *tlr3* and *vig1* responded similarly, showing a more pronounced upregulation from 72 h onwards. These findings are consistent with our previous study [22], where the peak of VHSV infection (72 h) in rainbow trout showed the strong expression of *irf3* and *7* and *vig1*, and also with the study by Krishman et al. [58], which observed the expression of these interferons in the brain of *Lates calcarifer* 48 h after infection with nervous necrosis virus. Furthermore, this overexpression functions as an antiviral response element in fish [59], reinforcing the importance of the skin and gills as protective mucosal barriers in viral infections.

### 4.3. Methionine Supplementation in Response to VHSV

Although dietary methionine supplementation did not significantly reduce viral copy numbers across all analysed tissues, trout fed with methionine (MET) exhibited a trend toward lower viral loads, particularly at the peak of infection (72 h). This observation suggests that a surplus of methionine may have the potential to modulate and support the host’s immune response to viral infection. This hypothesis is further supported by changes observed in systemic cellular and humoral activity and a transcriptional response indicative of improved viral recognition/signalling, inflammatory response, and direct antiviral activity.

Regardless of infection status, a higher dietary methionine supply led to reduced leukocyte recruitment at 120 h post-infection compared to the CTRL diet, which could initially suggest a dampened inflammatory response. However, when considering the data more comprehensively, the opposite appears to be true. Enhanced plasma peroxidase activity was observed not only after the feeding period and prior to any challenge (0 h) but also at 72 h in both the infected and uninfected groups and again at 120 h in the infected fish, indicating a strengthened immune response overall. The peroxidase enzymatic activity facilitates the use of ROS to eliminate pathogens [60]; therefore, the MET diet may contribute more effectively than the CTRL diet to VHSV clearance, partially via this mechanism, as evidenced by the tendency for a reduction in the viral load at the peak of infection observed in most tissues, although it is not significant. With that, the leukopenia observed at 120 h may reflect a scenario of a more rapid resolution of the infection.

When there is a VHSV infection through water, skin and gills are the external tissues that make direct contact with the virus, playing an important role in the dissemination of the pathogen, as they allow for the absorption and subsequent replication within the host [61,62]. In fact, Vaz et al. [22] recently described the gills and skin as the entry route of the virus in rainbow trout. Considering the importance of those mucosal surfaces [63,64], the transcriptional responses of skin and gills were analysed more thoroughly at the defined peak of infection—72 h. Given this, the genes regulated at the peak of infection in both tissues were investigated, and the GO terms enriched exclusively in response to the MET were analysed.

VHSV, a single-stranded, negative-sense RNA virus (ssRNA), is recognised and signalled by host cells via RIG-I-like receptors (RLRs), specifically mediated by retinoic acid-inducible gene I (RIG-I)/melanoma differentiation-associated gene 5 (MDA5). Toll-like receptors (TLRs) allow for endogenous recognition in downstream signalling cascades [65], which ultimately lead to the induction of an innate immune response via type I interferon (IFN I) [66,67]. Thereafter, this initial receptor’s molecules establish contact with the myeloid differentiation primary response 88 (MyD88) protein, which functions as a universal and important adaptor in various viral infections [68,69]. In this study, at the peak of infection, methionine dietary surplus induced the overexpression of *tlr3*—a TLR family member involved in the induction of type I interferons (IFNs), pro-inflammatory cytokines, and chemokines—as well as *myd88* in the skin and gills under the common pathway “positive regulation of immune system process.” In fact, qPCR results showed an improved mRNA expression of *myd88* in the gills as a response to dietary methionine treatment at 120 h. It is noteworthy that, in the gills, both genes appeared to be enriching a greater number of pathways, demonstrating that, at the peak of infection, the MET diet promoted the pronounced recognition/signalling of the VHSV presence, leading to a rapid induction of the type I IFN response.

After the initial recognition of the virus, there is a downstream cascade response with the activation of the mitochondrial antiviral signalling protein (MAVS), leading to an association with TNF receptor-associated factor 2 (TRAF 2), which, in turn, activates TANK Binding Kinase 1 (TBK1/TANK) [65,70], ultimately promoting phosphorylation in the serine-rich C-terminal region of the IFN regulatory factors 3 and 7 [66,71]. Additionally, TRAF can also activate nuclear factor kappa B (NF-κB) for signal transduction through other receptors like TLRs [72], regulating the expression of various genes involved in cell survival and inflammatory responses [72]. As a consequence of the higher methionine availability, *traf2* and *nfkb* genes were, in fact, found to be positively regulated in the skin or in both the skin and gills, respectively, at the peak of infection, according to RNA sequencing results, which may indicate a modulation of the mediated cell proliferation, possibly through the synthesis of polyamines via the aminopropylation pathway involved in the methionine cycle, inflammation, and the expression pathways [73,74] involved in host response to combat the infection [64,75]. These findings also suggest that the diet supplemented with this essential amino acid appears to promote a controlled and effective response through the upregulation of potent inflammatory pathways and mechanisms involved in cell death.

Similarly to MyD88, the stimulator of interferon genes (STING) functions as a viral adaptor directly activated by the presence of viral components within the cell [73,76,77]. Upon activation, STING binds to TBK1, promoting the phosphorylation of IRF3/7 and NF-κB, which then translocate to the nucleus and bind to interferon-stimulated genes, ultimately inducing the expression of type I interferons and pro-inflammatory cytokines [73,78,79]. In this study, significant positive expression of this adaptor (STING) was observed in the MET diet at 72 h in both tissues, in various pathways, including those related to type I interferon production, further confirming and highlighting its essential role in the early production of an antiviral response in fish, as observed by Feng et al. [80] and Li et al. [73]. Such results were confirmed via qPCR in skin, with a significant upregulation of this gene in MET-fed fish compared to the CTRL group at 72 h post-infection. Additionally, enhanced activation of the transcription factors *irf3* and *irf7* in response to VHSV was observed in both the skin and gills following methionine supplementation. This was evident through both transcriptome-wide analysis and targeted qPCR validation [19,59]. Additionally, the incidence of a virus also induces the expression of various elements involved in the pro-inflammatory cytokine responses of the acute phase, such as tumour necrosis factor α, which plays a role in regulating inflammation and cellular apoptosis [2,4]. A significantly elevated induction of this gene (*tnfα*) was observed at the peak of the infection in the gills of trout fed MET, significantly enriching pathways involved in the “regulation of immune system process/cellular process”. This indicates an improved inflammatory action in this mucosa in response to the dietary treatment, as well as a possible regulation of cellular apoptosis due to the high incidence of VHSV at this time.

Finally, once the activation pathways reach the cell’s nucleus, the translation into protein of some interferon-stimulated genes (ISGs), such as Myxovirus (Mx), viperin/RSAD2, and interferon stimulated genes 15 (ISG15), is promoted and, once bonded, IRF3/7 are activated [21,71,81], acting directly to reduce and degrade the virus [66,82,83]. Methionine dietary surplus positively regulated viperin (*vig1/rsad2*) in the gills, specifically as a cellular component associated with the cytoplasmic pathway—likely the primary site of the host response prior to translocation into the nucleus, where the innate immune response is activated. Many studies have focused on and highlighted the importance of viperin activity due to its broad-spectrum antiviral capability across various species and cell types, especially in negative-strand RNA viruses [22,81,84,85]. This antiviral protein houses three N-terminal domains, a radical S-adenosyl-L-methionine (SAM) domain, and a C-terminal domain [81,86]. The SAM domain is functionally linked to the cytoplasmic methionine (Met) synthesis cycle in fish cells and supports SAM-dependent cellular processes; rather than acting as a classical methyltransferase, the radical SAM domain catalyses radical-based reactions via the generation of a 5′-deoxyadenosyl radical, thereby modulating gene transcription and expression [6,87]. In the gills, *vig1*/*rsad2* expression was significantly higher in fish fed the MET diet, coinciding with the heightened VHSV activity observed in the present study. This further confirms the direct action of this protein against VHSV, as previously observed in rainbow trout [21,22], redlip mullet (*Liza haematocheila*) [81,88], and zebra fish (*Danio rerio*) [89], as well as the ability of methionine’s higher dietary availability to further induce its transcription. Given this, the pronounced upregulation of *vig1*, a SAM-dependent antiviral protein, in methionine-fed fish appears to provide a direct molecular link between nutritional intervention and the enhanced antiviral state, suggesting a possible priming of the SAM metabolic pool for immune protein synthesis.

It was noted that methionine dietary supplementation also contributed to a significant increase in several caspase expressions. In addition to triggering antiviral proteins, caspases and cathepsins act to maintain homeostasis by regulating the number of cells in tissues in infection scenarios and are responsible for programmed cell death when homeostasis needs to be maintained [90,91]. In addition to their roles in cell proliferation and differentiation, they are also involved in reducing viral replication [91]. Specifically in the gills, significant upregulation of *caspase-1*, *3*, *8*, and *14* was observed, enriching pathways related to cysteine (“cysteine-type peptidase/endopeptidase activity”). Cathepsins (B, C, H, L, and K) were also detected in the same pathways as caspases and were significantly expressed under the GO term “cytoplasm”.

In summary, the present study explores the role of methionine during the development of an inflammatory response caused by VHSV. At the defined peak of infection, methionine was shown to improve the rainbow trout immune response to VHSV, supported by the modulation of multiple pathways related to the methionine cycle. The external mucosa of the fish, the skin and gills, seem to function as the first barrier to the entry of infectious agents, displaying the upregulation of pathways related to the general immune system. Gill response stood out due to the strong upregulation of genes and the enrichment of various pathways related to DNA methylation. Also, methionine dietary supplementation allowed for the improved response of trout against VHSV infection, promoting the positive activation and significantly higher expression of important antiviral genes (e.g., *irf3*, -7, and *vig1*), improving the host’s response at the peak of infection. On the other hand, the absence of such pronounced systemic effects is likely due to the nature of the infection, which was designed to mimic a more natural viral bath exposure. This route of infection tends to elicit a stronger localised response, particularly at mucosal sites, compared to the systemic response. Indeed, recent studies [25] provide support for the hypothesis that, during a bacterial bath infection, methionine elicited only a modest systemic effect while concurrently inducing a pronounced mucosal response.

## 5. Conclusions

This study demonstrated that increasing the amount of methionine in the rainbow trout diet to twice the nutritional requirement for 4 weeks improved the antiviral response against VHSV, corroborating the previously described modulatory capacity of methionine. Such a modulatory capacity was observed not only at the systemic level but also at tissue level, with both the gills and skin exhibiting the significant upregulation of GO terms associated with an improved immune response. The present findings support the use of methionine supplementation as a strategy to improve the local and systemic response of fish to viral infections in a prophylactic manner, contributing to the improvement of fish health and welfare.

## Figures and Tables

**Table 1:** Ingredients and chemical composition of the CTRL and MET experimental diets.

Ingredients	%	
	CTRL	MET
Fishmeal super prime ^1^	20.00	20.00
Poultry meal ^2^	7.50	7.50
Poultry blood meal ^3^	2.00	2.00
Soy protein concentrate ^4^	18.00	18.00
Wheat gluten ^5^	12.00	11.00
Corn gluten meal ^6^	8.00	8.00
Wheat meal ^7^	9.90	9.90
Faba beans (low tannins) ^8^	5.00	5.00
Vitamin and mineral premix PV01 ^9^	1.00	1.00
Antioxidant ^10^	0.20	0.20
MCP (monocalcium phosphate) ^11^	1.00	1.00
Fish oil ^12^	5.00	5.00
Rapeseed oil ^13^	10.40	10.40
DL-Methionine ^14^	**0.00**	**1.00**
Total	100	100
**Proximate Analysis (% dry weight)**		
Ash (g/100 g)	7.85	7.83
Protein (g/100 g)	50.75	50.97
Fat (g/100 g)	19.12	19.19
Phosphor (g/100 g)	1.05	1.01
Energy (KJ/g)	22.32	22.43

^1^ Fishmeal Super Prime: 66.3% CP, 11.5% CF, Pesquera Diamante, Callao, Peru. ^2^ Poultry meal: 62.4% CP, 14.5% CF, SAVINOR UTS, Trofa, Portugal. ^3^ Poultry blood meal: 90% CP, 1%CF, ECB COMPANY SRL A S.U, Treviglio, Italy. ^4^ Soycomil P: 63% CP, 0.8% CF, ADM, Amsterdam, The Netherlands. ^5^ VITAL: 83.7% CP, 1.6% CF, ROQUETTE Frères, Lestrem, France. ^6^ Corn gluten meal: 61% CP, 6% CF, COPAM, São João da Talha, Portugal. ^7^ Wheat meal: 10.2% CP, 1.2% CF, Casa Lanchinha, Alhos Vedros, Portugal. ^8^ Faba beans: 28% CP, 1.2% CF, Ribeiro & Sousa Lda, Lisboa, Portugal. ^9^ PREMIX Lda, Vila do Conde, Portugal: Vitamins (IU or mg/kg diet): dl-alpha tocopherol acetate, 100 mg; sodium menadione bisulphate, 25 mg; retinyl acetate, 20,000 IU; dl-cholecalciferol, 2000 IU; thiamin, 30 mg; riboflavin, 30 mg; pyridoxine, 20 mg; cyanocobalamin, 0.1 mg; nicotinic acid, 200 mg; folic acid, 15 mg; ascorbic acid, 500 mg; inositol, 500 mg; biotin, 3 mg; calcium panthotenate, 100 mg; choline chloride, 1000 mg; and betaine, 500 mg. Minerals (g or mg kg^−1^ diet): copper sulphate, 9 mg; ferric sulphate, 6 mg; potassium iodide, 0.5 mg; manganese oxide, 9.6 mg; sodium selenite, 0.01 mg; zinc sulphate, 7.5 mg; sodium chloride, 400 mg; and excipient wheat middlings. ^10^ Paramega PX, Kemin Europe NV, Herentals, Belgium. ^11^ MCP (monocalcium phosphate): 22% phosphorus, 16% calcium, Fosfitalia, Italy. ^12^ SAVINOR UTS, Trofa, Portugal. ^13^ Henry Lamotte Oils GmbH, Bremen, Germany. ^14^ DL-Methionine for aquaculture: 99% methionine, Evonik Nutrition & Care GmbH, Essen, Germany.

**Table 2:** Amino acid composition (g AA 100 g^−1^ dry weight) of experimental diets: CTRL and MET.

Amino Acids (g AA 100 g^−1^ DW)	Experimental Diets	
	CTRL	MET
Arginine	3.00	2.92
Histidine	1.10	1.06
Isoleucine	1.90	2.00
Leucine	4.10	3.65
Lysine	2.60	2.88
Threonine	1.80	1.84
Valine	2.20	2.25
**Methionine**	**0.80**	**1.78**
Cysteine	0.60	0.60
Methionine + Cysteine	1.40	2.38
Phenylalanine	2.30	2.23
Tyrosine	1.70	1.47
Phenylalanine + Tyrosine	4.10	3.7
Taurine	0.10	0.10
Aspartic acid + Asparagine	4.20	4.34
Glutamic acid + Glutamine	9.20	9.57
Alanine	2.60	2.60
Glycine	2.70	3.21
Proline	3.40	3.30
Serine	2.30	2.35

**Table 3:** Genes analysed in skin and gills of rainbow trout by real-time PCR.

Gene	Acronym	Accession Number	Efficiency (%)		Annealing Temperature (°C)		Product Length (bp)	Forward	Reserve	Reference
			Skin	Gills	Skin	Gills				
Elongation factor 1 alpha	*ef1α*	NM_001124339.1	125	105	55		159	GGCAAGTCAACCACCACAG	GATACCACGCTCCCTCTCAG	-
B-actin	*actβ*	AF157514.1	94	99	58		260	ATGGAAGGTGAAATCGCC	TGCCAGATCTTCTCCATG	-
18s ribosomal RNA	*18s*	FJ710874.1	89	101	60	62	147	GGCCGTTCTTAGTTGGTGGA	TTGCTCAATCTCGTGTGGCT	-
Radical S-adenosyl methionine domain containing 2/viperin	*vig1*	XM_021582972.2	91	93	60	62	204	GACAGCTTTGACGAGGACAC	GAACACCTTCCAGCGTACAG	-
Interferon regulatory factor 3	*irf3*	NM_001257262.1	116	106	62		143	ATGAACAGCGGATGGTGGAG	GAACCCTGCCTCGTTCTCAA	-
Interferon regulatory factor 7	*irf7*	AJ829673.1	114	93	62		317	ATGGTTACCGGCAGGACAAG	GTCATTCTGGAGGGACGACG	-
Toll-like receptor 3	*tlr3*	NM_001124578	91	92	62		61	AGCCCTTTGCTGCCTTACAGAG	GTCTTCAGGTCATTTTTGGACACG	[36]
MYD88 innate immune signal transduction adaptor	*myd88*	NM_001124421.1	93	92	62		157	CGGGTGCTCTCCTCCTTTTG	CCATATGAGCGATGGGGCTC	-
Stimulator of interferon genes protein-like	*sting*	XM_021559672.2	96	101	62		186	GGTGGGACAGGTCTAAGGAGA	GAGAACCCGAACATCCAGTG	-
Tumour necrosis factor α	*tnfα*	AJ277604, AJ401377	102	94	55		75	GGGGACAAACTGTGGACTGA	GAAGTTCTTGCCCTGCTCTG	[37]

**Table 4:** White blood cells (WBC) (×10^4^ µL^− 1^), red blood cells (RBC) (×10^6^ µL^− 1^), haematocrit (HT) (%), mean corpuscular volume (MCV) (µm^3^), haemoglobin (HG) (g dL^−1^), mean corpuscular haemoglobin (MCH) (pg cell^−1^), and mean corpuscular haemoglobin concentration (MCHC) (g 100 mL^−1^) of rainbow trout fed with CTRL and MET diets, exposed to a challenge with VHSV, and sampled at 0, 24, 72, and 120 h post-challenge.

Parameters	0 h		Non-Infected						Infected					
	CTRL	MET	CTRL			MET			CTRL			MET		
			24 h	72 h	120 h	24 h	72 h	120 h	24 h	72 h	120 h	24 h	72 h	120 h
WBC	1.72 ± 0.91	1.94 ± 1.06	0.81 ± 0.2	0.87 ± 0.1	1.34 ± 0.6	0.89 ± 0.37	0.82 ± 0.24	0.63 ± 0.27	0.97 ± 0.26	0.59 ± 0.23	1.83 ± 0.57	0.88 ± 0.39	0.59 ± 0.17	1.40 ± 0.56
RBC	0.89 ± 0.13	0.88 ± 0.10	0.67 ± 0.16	1.07 ± 0.34	1.86 ± 0.58	0.78 ± 0.10	1.19 ± 0.40	1.91 ± 0.61	0.81 ± 0.12	1.09 ± 0.21	0.88 ± 0.33	0.79 ± 0.16	1.09 ± 0.29	1.16 ± 0.40
HT	32.29 ± 4.94	35.00 ± 2.78	34.33 ± 3.84	36.00 ± 8.83	38.57 ± 3.69	35.67 ± 2.40	39.67 ± 3.72	41.17 ± 4.60	33.33 ± 4.09	32.22 ± 3.73	29.50 ± 7.19	34.89 ± 4.63	34.00 ± 3.62	33.50 ± 0.87
MCV	369.43 ± 63.46	406.77 ± 50.90	511.45 ± 164.81	376.56 ± 155.63	206.16 ± 87.91	463.18 ± 68.79	320.84 ± 83.13	221.91 ± 81.47	415.10 ± 32.43	311.69 ± 71.25	257.21 ± 33.78	458.66 ± 101.77	330.44 ± 70.49	275.29 ± 119.54
HG	2.04 ± 0.64	2.16 ± 0.70	3.18 ± 3.94	1.21 ± 0.49	3.31 ± 1.70	1.84 ± 0.62	1.94 ± 0.39	2.01 ± 0.27	1.70 ± 0.39	1.85 ± 0.58	1.31 ± 0.57	1.91 ± 0.68	1.53 ± 0.49	1.93 ± 0.83
MCH	22.73 ± 5.90	23.38 ± 6.10	25.99 ± 8.46 ^a^	11.53 ± 3.42 ^b^	14.71 ± 7.67 ^b^	24.11 ± 9.68 ^a^	19.33 ± 9.61 ^b^	10.23 ± 3.17 ^b^	21.01 ± 3.55 ^a^	17.23 ± 4.00 ^b^	12.96 ± 5.63 ^b^	24.07 ± 5.79 ^a^	14.13 ± 3.98 ^b^	15.79 ± 5.34 ^b^
MCHC	6.42 ± 1.79	5.57 ± 1.76	5.17 ± 1.42	3.34 ± 1.07	9.67 ± 4.99	5.17 ± 1.68	4.86 ± 0.78	4.95 ± 0.85	5.11 ± 1.00	5.90 ± 2.20	5.68 ± 1.24	5.44 ± 1.62	4.45 ± 1.32	6.29 ± 2.40

Values represent means ± SD (*n* = 16 for 0 h; *n* = 9 for 24, 72, and 120 h). Different lowercase letters indicate differences between times (0, 24, 72, and 120 h). Multifactorial ANOVA; Holm–Šídák post hoc test; *p* ≤ 0.05. CTRL (control diet), MET (methionine diet).

**Table 5:** Main genes and upregulated genes in the skin and gills and respective pathways exclusively enriched in response to the MET diet at the peak of infection—72 h. The pathways were categorised by function. BP: biological process; CC: cellular component; and MF: molecular function. TLR3: Toll-like receptor 3; MyD88: Myeloid differentiation primary response 88; TRAF2: tumour necrosis factor (TNF) receptor associated 2; NF κB: nuclear factor kappa B; STING: stimulator of interferon genes; IRF (3 and 7): interferon regulatory factors 3 and 7; VIG1/RSAD2: viperin/Radical S-Adenosyl Methionine Domain Containing 2; CASP: caspase; CTS: cathepsin; and TNFα: tumour necrosis factor α.

Differentially Upregulated Genes	Skin		Gills	
	Function	Enriched Pathways	Function	Enriched Pathways
**TLR3**	BP	positive regulation of immune system process	BP	regulation of immune system process
				positive regulation of immune system process
				pattern recognition receptor signalling pathway
				immune response-regulating signalling pathway
				immune response-activating signalling pathway
**MyD88**	BP	positive regulation of immune system process	BP	regulation of immune system process
		intracellular signal transduction		regulation of immune response
				positive regulation of immune system process
				positive regulation of cellular process
				pattern recognition receptor signalling pathway
				immune response-regulating signalling pathway
				immune response-activating signalling pathway
**TRAF2**	BP	intracellular signal transduction	-	
**NF** **κB**	BP	regulation of gene expression	CC	cytoplasm
**STING**	BP	type I interferon production	CC	cytoplasm
		regulation of type I interferon production	BP	type I interferon production
		regulation of gene expression		regulation of immune system process
		positive regulation of type I interferon production		regulation of immune response
				positive regulation of immune system process
				positive regulation of cellular process
**IRF3**	BP	regulation of gene expression	MF	transcription regulatory region nucleic acid binding
			BP	positive regulation of cellular process
**IRF7**	BP	regulation of gene expression	MF	transcription regulatory region nucleic acid binding
			BP	positive regulation of cellular process
**VIG1/RSAD2**	-		CC	cytoplasm
**CASP**	-		MF	cysteine-type peptidase activity
				cysteine-type endopeptidase activity
**CTS**	-		MF	cysteine-type peptidase activity
			CC	cytoplasm
**TNFα**	-		CC	cytoplasm
			BP	regulation of immune system process
				positive regulation of cellular process

## Data Availability

The data used in this study are available in public and online repositories. Details about the repository(ies) and corresponding accession number(s) are available at https://doi.org/10.6084/m9.figshare.30209674.v1 and https://doi.org/10.6084/m9.figshare.30209632.v1.

## Supplementary Materials

The following supporting information can be downloaded at: https://www.mdpi.com/article/10.3390/biology15020163/s1, Figure S1: Venn diagram showing the number of common or unique genes related to the CTRL and MET diet resulting from Gene Ontology (GO) analysis, which were upregulated (A) or downregulated (B) in the skin at 72 h post-VHSV challenge; Figure S2: Venn diagram showing the number of common or unique genes related to the CTRL and MET diet resulting from Gene Ontology (GO) analysis, which were upregulated (A) or downregulated (B) in the gills at 72 h post-VHSV challenge: Table S1: *p*-values from one-way ANOVA and multifactorial ANOVA for haematological profile of rainbow trout fed with the CTRL and MET diets, exposed to a VHSV-challenge, and sampled at 0, 24, 72, and 120 h after infection. Different lowercase letters indicate differences between times (0, 24, 72, and 120 h) and uppercase letters indicate differences between infection (non-infected vs. infected). Different symbols represent differences between experimental diets (CTRL vs. MET). (ns: non-significant); Table S2: *p*-values from one-way ANOVA and multifactorial ANOVA for differential leukocytes in the blood of rainbow trout fed with the CTRL and MET diets, exposed to a VHSV-challenge, and sampled at 0, 24, 72, and 120 h after infection. Different lowercase letters indicate differences between times (0, 24, 72, and 120 h) and uppercase letters indicate differences between infection (non-infected vs. infected). Different symbols represent differences between experimental diets (CTRL vs. MET). (ns: non-significant); Table S3: *p*-values from one-way ANOVA and multifactorial ANOVA for humoral immune response in plasma of rainbow trout fed with the CTRL and MET diets, exposed to a VHSV-challenge, and sampled at 0, 24, 72, and 120 h after infection. Different lowercase letters indicate differences between times (0, 24, 72, and 120 h) and uppercase letters indicate differences between infection (non-infected vs. infected). Different symbols represent differences between experimental diets (CTRL vs. MET). (ns: non-significant); Table S4: *p*-values from one-way ANOVA and multifactorial ANOVA for biomarkers of oxidative stress in liver of rainbow trout fed with the CTRL and MET diets, exposed to a VHSV-challenge, and sampled at 0, 24, 72, and 120 h after infection. Different lowercase letters indicate differences between times (0, 24, 72, and 120 h) and uppercase letters indicate differences between infection (non-infected vs. infected). Different symbols represent differences between experimental diets (CTRL vs. MET). (ns: non-significant); Table S5: *p*-values from multifactorial ANOVA for viral load in gills, skin, liver, head-kidney and spleen of rainbow trout fed with the CTRL and MET diets, exposed to a VHSV challenge, and sampled at 0, 24, 72, and 120 h after infection. Different lowercase letters indicate differences between times (0, 24, 72, and 120 h) and uppercase letters indicate differences between infection (non-infected vs. infected). Different symbols represent differences between experimental diets (CTRL vs. MET). (ns: non-significant); Table S6: *p*-values from one-way ANOVA and multifactorial ANOVA for gene expression in skin of rainbow trout fed with the CTRL and MET diets, exposed to a VHSV-challenge, and sampled at 0, 24, 72, and 120 h after infection. Different lowercase letters indicate differences between times (0, 24, 72, and 120 h) and uppercase letters indicate differences between infection (non-infected vs. infected). Different symbols represent differences between experimental diets (CTRL vs. MET). (ns: non-significant); Table S7: *p*-values from one-way ANOVA and multifactorial ANOVA for gene expression in gills of rainbow trout fed with the CTRL and MET diets, exposed to a VHSV-challenge, and sampled at 0, 24, 72, and 120 h after infection. Different lowercase letters indicate differences between times (0, 24, 72, and 120 h) and uppercase letters indicate differences between infection (non-infected vs. infected). Different symbols represent differences between experimental diets (CTRL vs. MET). (ns: non-significant).
