# Supplementary material for: Dietary Methionine Supplementation Improves Rainbow Trout (Oncorhynchus mykiss) Immune Responses Against Viral Haemorrhagic Septicaemia Virus (VHSV)
Source: Biology (Basel). 2026 Jan 16;15(2):163. doi: 10.3390/biology15020163 (PMC12837368; doi:10.3390/biology15020163)
Supplement: Supplementary file 1 [file biology-15-00163-s001.zip › biology-4074205-supplementary.pdf]

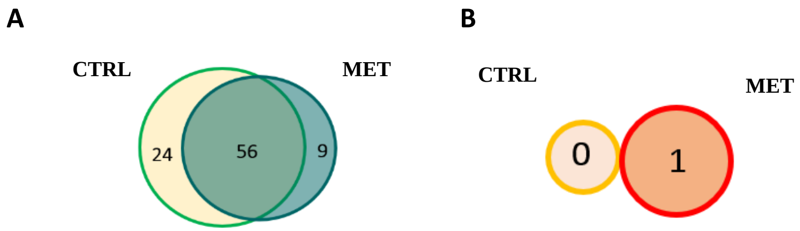

Figure S1. Venn diagram showing the number of common or unique genes related to the CTRL and MET diet resulting from Gene Ontology (GO) analysis, which were upregulated **(A)** or downregulated **(B)** in the skin at 72 h post-VHSV challenge.

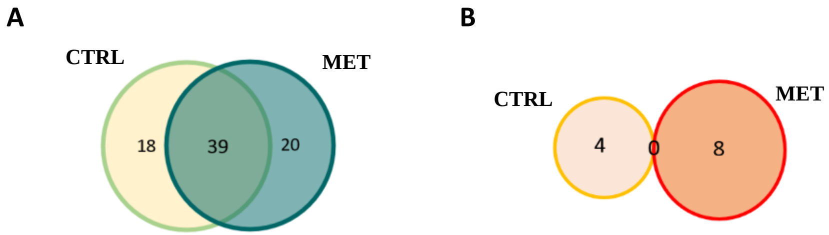

Figure S2. Venn diagram showing the number of common or unique genes related to the CTRL and MET diet resulting from Gene Ontology (GO) analysis, which were upregulated **(A)** or downregulated **(B)** in the gills at 72 h post-VHSV challenge.

Table S1: P-values from one-way ANOVA and multifactorial ANOVA for haematological profile of rainbow trout fed with the CTRL and MET diets, exposed to a VHSV-challenge, and sampled at 0, 24, 72, and 120 h after infection. Different lowercase letters indicate differences between times (0, 24, 72, and 120 h) and uppercase letters indicate differences between infection (non-infected vs infected). Different symbols represent differences between experimental diets (CTRL vs MET). (ns: non-significant).

Parameters	One-way ANOVA	Multifactorial ANOVA														Time x Diet						Time x Infection					
	Diet	Time	Diet	Infection	Time x Diet	Time x Infection	Infection x Diet	Time x Diet x Infection	Time			Diet		Infection		CTRL			MET			Non-infected			Infected		
									24h	72h	120h	CTRL	MET	Non-infected	Infected	24h	72h	120h	24h	72h	120h	24h	72h	120h	24h	72h	120h
WBC	ns	<0.001	0.001	0.024	0.029	<0.001	ns	ns	-	-	-	-	-	-	-	-	-	*	-	-	#	-	A	B	-	B	A
RBC	ns	<0.001	ns	<0.001	ns	<0.001	ns	ns	-	-	-			-	-	-	-	-	-	-	-	-	-	A	-	-	B
HT	ns	ns	ns	<0.001	ns	0.006	ns	ns	-	-	-	-	-	-	-	-	-	-	-	-	-	-	A	A	-	B	B
MCV	ns	<0.001	ns	ns	ns	ns	ns	ns	a	b	c	-	-	-	-	-	-	-	-	-	-	-	-	-	-	-	-
HG	ns	ns	ns	ns	ns	ns	ns	ns	-	-	-	-	-	-	-	-	-	-	-	-	-	-	-	-	-	-	-
MCH	ns	<0.001	ns	ns	ns	ns	ns	0.013	-	-	-	-	-	-	-	-	-	-	-	-	-	-	-	-	-	-	-
MCHC	ns	0.021	ns	ns	ns	ns	ns	ns	a	b	a	-	-	-	-	-	-	-	-	-	-	-	-	-	-	-	-

Table S2: P-values from one-way ANOVA and multifactorial ANOVA for differential leukocytes in the blood of rainbow trout fed with the CTRL and MET diets, exposed to a VHSV-challenge, and sampled at 0, 24, 72, and 120 h after infection. Different lowercase letters indicate differences between times (0, 24, 72, and 120 h) and uppercase letters indicate differences between infection (non-infected vs infected). Different symbols represent differences between experimental diets (CTRL vs MET). (ns: non-significant).

Parameters	One-way ANOVA	Multifactorial ANOVA							Time x Infection								
	Diet	Time	Diet	Infection	Time x Diet	Time x Infection	Infection x Diet	Time x Diet x Infection	Time			NV			V		
									24	72	120	24	72	120	24	72	120
Lymphocytes	ns	**0.013**	ns	ns	ns	**0.020**	ns	ns	-	-	-	-	-	B	-	-	A
Thrombocytes	ns	ns	ns	ns	ns	**0.010**	ns	ns	-	-	-	-	-	A	-	-	B

Neutrophils	ns	ns	ns	ns	ns	ns	ns	ns	-	-	-	-	-	-	-	-	-
Monocytes	ns	**<0.001**	ns	ns	ns	ns	ns	ns	b	c	a	-	-	-	-	-	-

Table S3: P-values from one-way ANOVA and multifactorial ANOVA for humoral immune response in plasma of rainbow trout fed with the CTRL and MET diets, exposed to a VHSV-challenge, and sampled at 0, 24, 72, and 120 h after infection. Different lowercase letters indicate differences between times (0, 24, 72, and 120 h) and uppercase letters indicate differences between infection (non-infected vs infected). Different symbols represent differences between experimental diets (CTRL vs MET). (ns: non-significant).

Parameters	One-way ANOVA	Multifactorial ANOVA							Time x Infection												
									Time			Diet		Infection		NV			V		
	Diet	Time	Diet	Infection	Time x Diet	Time x Infection	Infection x Diet	Time x Diet x Infection	24	72	120	CTRL	MET	NV	V	24	72	120	24	72	120
Antiproteases	ns	ns	ns	ns	ns	ns	ns	ns	-	-	-	-	-	-	-	-	-	-	-	-	-
Proteases	ns	ns	ns	ns	ns	ns	ns	ns	-	-	-	-	-	-	-	-	-	-	-	-	-
Lysozyme	ns	<0.001	ns	0.008	ns	**0.004**	ns	ns	-	-	-	-	-	A	B	-	A	A	-	B	B
Peroxidase	**0.038**	<0.001	0.014	<0.001	ns	**0.02**	ns	ns	-	-	-	*	#	A	B	-	-	A	-	-	B
Nitric oxide	ns	<0.001	ns	0.021	ns	**0.038**	ns	ns	-	-	-	-	-	A	B	-	-	A	-	-	B

Table S4: P-values from one-way ANOVA and multifactorial ANOVA for biomarkers of oxidative stress in liver of rainbow trout fed with the CTRL and MET diets, exposed to a VHSV-challenge, and sampled at 0, 24, 72, and 120 h after infection. Different lowercase letters indicate differences between times (0, 24, 72, and 120 h) and uppercase letters indicate differences between infection (non-infected vs infected). Different symbols represent differences between experimental diets (CTRL vs MET). (ns: non-significant).

Parameters	One-way ANOVA	Multifactorial ANOVA							Time x Infection										
									Time			Infection		NV			V		
	Diet	Time	Diet	Infection	Time x Diet	Time x Infection	Infection x Diet	Time x Diet x Infection	24	72	120	NV	V	24	72	120	24	72	120
SOD	ns	<0.001	ns	0.008	ns	**<0.001**	ns	ns	-	-	-	-	-	A	-	-	B	-	-

CAT	ns	**<0.001**	ns	**<0.00 1**	ns	ns	ns	ns	a	b	a	A	B	A	B	A	A	B	A
GST	ns	<0.001	ns	0.027	ns	**<0.001**	ns	ns	-	-	-	-	-	B	B	B	A	A	A
Ratio GSH/GSSG	ns	**<0.001**	ns	ns	ns	ns	ns	ns	a	a	b	-	-	-	-	-	-	-	-

Table S5: P-values from multifactorial ANOVA for viral load in gills, skin, liver, head-kidney and spleen of rainbow trout fed with the CTRL and MET diets, exposed to a VHSV-challenge, and sampled at 0, 24, 72, and 120 h after infection. Different lowercase letters indicate differences between times (0, 24, 72, and 120 h) and uppercase letters indicate differences between infection (non-infected vs infected). Different symbols represent differences between experimental diets (CTRL vs MET). (ns: non-significant).

Parameters	Multifactorial ANOVA					
				Time		
	Time	Diet	Time x Diet	24	72	120
Gills	**<0.001**	ns	ns	c	a	b
Skin	**0.002**	ns	ns	c	a	b
Liver	**0.05**	ns	ns	b	a	a
Head-kidney	**<0.001**	ns	ns	c	a	b
Spleen	**0.05**	ns	ns	b	a	a

Table S6: P-values from one-way ANOVA and multifactorial ANOVA for gene expression in skin of rainbow trout fed with the CTRL and MET diets, exposed to a VHSV-challenge, and sampled at 0, 24, 72, and 120 h after infection. Different lowercase letters indicate differences between times (0, 24, 72, and 120 h) and uppercase letters indicate differences between infection (non-infected vs infected). Different symbols represent differences between experimental diets (CTRL vs MET). (ns: non-significant).

Parameters	One-way ANOVA	Multifactorial ANOVA														Time x Diet						Time x Infection						Infection x Diet				Time x Diet x Infection											
	Diet	Time	Diet	Infection	Time x Diet	Time x Infection	Infection x Diet	Time x Diet x Infection	Time			Diet		Infection		CTRL			MET			NV			V			CTRL		MET		CTRL						MET					
																																24		72		120		24		72		120	
									24	72	120	CTRL	MET	NV	V	24	72	120	24	72	120	24	72	120	24	72	120	NV	V	NV	V	NV	V	NV	V	NV	V	NV	V	NV	V	NV	V
Tlr3	0.002	<0.001	ns	<0.001	0.009	<0.001	ns	**0.003**	-	-	-	-	-	-	-	ab	c	c	ab	c	c	-	-	-	-	-	-	-	-	-	-	B	B	B	A	B	A	B	B	B	A	B	A
Myd88	ns	<0.001	ns	<0.001	ns	**0.007**	ns	ns	-	-	-	-	-	-	-	-	-	-	-	-	-	-	-	-	c	a	b	-	-	-	-	.	.	-	-	-	-	-	-	-	-	-	-
Sting	ns	<0.001	0.015	<0.001	0.002	<0.001	0.020	**0.005**	-	-	-	-	-	-	-	-	-	-	a	b	c	-	-	-	a	b	c	-	-	B	A	-	-	-	*	-	-	-	-	B	A#	-	-
Irf3	ns	<0.001	ns	<0.001	ns	ns	ns	**0.037**	-	-	-	-	-	-	-	-	-	-	-	-	-	-	-	-	-	-	-	-	-	-	-	-	-	-	-	-	-	-	-	B	A	-	-
Irf7	ns	<0.001	ns	<0.001	ns	**0.001**	ns	ns	-	-	-	-	-	-	-	-	-	-	-	-	-	-	-	B	-	-	A	-	-	-	-	-	-	-	-	-	-	-	-	-	-	-	-
Vig1	0.022	0.002	ns	<0.001	ns	**0.002**	ns	ns	-	-	-	-	-	-	-	-	-	-	-	-	-	-	B	B	-	A	A	-	-	-	-	-	-	-	-	-	-	-	-	-	-	-	-
Tnfα	ns	**<0.001**	ns	**0.007**	ns	ns	ns	ns	a	b	a	-	-	A	B	-	-	-	-	-	-	-	-	-	-	-	-	-	-	-	-	-	-	-	-	-	-	-	-	-	-	-	-

Table S7: P-values from one-way ANOVA and multifactorial ANOVA for gene expression in gills of rainbow trout fed with the CTRL and MET diets, exposed to a VHSV-challenge, and sampled at 0, 24, 72, and 120 h after infection. Different lowercase letters indicate differences between times (0, 24, 72, and 120 h) and uppercase letters indicate differences between infection (non-infected vs infected). Different symbols represent differences between experimental diets (CTRL vs MET). (ns: non-significant).

Parameters	One-way ANOVA	Multifactorial ANOVA							Time x Diet														Time x Infection						Infection x Diet				Time x Diet x Infection											
									Time			Diet		Infection		CTRL			MET			NV			V			CTRL		MET		CTRL						MET					
																																24		72		120		24		72		120	
	Diet	Time	Diet	Infection	Time x Diet	Time x Infection	Infection x Diet	Time x Diet x Infection	24	72	120	CTRL	MET	NV	V	24	72	120	24	72	120	24	72	120	24	72	120	NV	V	NV	V	NV	V	NV	V	NV	V	NV	V	NV	V	NV	V
Tlr3	ns	<0.001	ns	<0.001	ns	**0.002**	ns	ns	-	-	-	-	-	-	-	-	-	-	-	-	-	-	B	B	-	A	A	-	-	-	-	-	-	-	-	-	-	-	-	-	-	-	-
Myd88	ns	ns	0.044	ns	0.044	0.011	0.002	**0.024**	-	-	-	-	-	-	-	-	-	-	b	b	a	-	-	-	-	-	-	-	*	-	#	-	-	-	-	-	-	-	b	-	b	-	a#
Sting	ns	0.003	ns	0.001	ns	**0.028**	**0.007**	**ns**	-	-	-	-	-	-	-	-	-	-	-	-	-	-	B	B	-	A	A	-	*	-	#	-	-	-	-	-	-	-	-	-	-	-	-
Irf3	ns	<0.001	ns	0.001	ns	**<0.001**	ns	ns	-	-	-	-	-	-	-	a	b	b	ab	b	ab	-	-	B	-	-	A	-	-	-	-	-	-	-	-	-	-	-	-	-	-	-	-
Irf7	ns	<0.001	<0.001	<0.001	**0.018**	**<0.001**	**0.03**	ns	-	-	-	-	-	-	-	a	ab	b	a	b	c	-	B	B	-	A	A	-	*	-	#	-	-	-	-	-	-	-	-	-	-	-	-
Vig1	ns	<0.001	0.017	<0.001	ns	**<0.001**	**0.019**	ns	-	-	-	-	-	-	-	-	-	-	-	-	-	-	B	B	-	A	A	-	*	-	#	-	-	-	-	-	-	-	-	-	-	-	-
Tnfα	ns	**<0.001**	ns	ns	ns	ns	ns	ns	a	b	b	-	-	-	-	-	-	-	-	-	-	-	-	-	-	-	-	-	-	-	-	-	-	-	-	-	-	-	-	-	-	-	-
